# Temperature-related mortality and associated vulnerabilities: evidence from Scotland using extended time-series datasets

**DOI:** 10.1186/s12940-022-00912-5

**Published:** 2022-10-25

**Authors:** Kai Wan, Zhiqiang Feng, Shakoor Hajat, Ruth M. Doherty

**Affiliations:** 1 School of GeoSciences, University of Edinburgh, Edinburgh, UK; 2 Scottish Centre for Administrative Data Research, School of Geosciences, University of Edinburgh, Edinburgh, UK; 3 Department of Public Health, Environments and Society, London School of Hygiene & Tropical Medicine, London, UK; 4 Centre On Climate Change and Planetary Health, London School of Hygiene & Tropical Medicine, London, UK

**Keywords:** Extreme cold, Extreme heat, Mortality risk, Vulnerability, Temporal change, Scotland

## Abstract

**Background:**

Adverse health impacts have been found under extreme temperatures in many parts of the world. The majority of such research to date for the UK has been conducted on populations in England, whilst the impacts of ambient temperature on health outcomes in Scottish populations remain largely unknown.

**Methods:**

This study uses time-series regression analysis with distributed lag non-linear models to characterise acute relationships between daily mean ambient temperature and mortality in Scotland including the four largest cities (Aberdeen, Dundee, Edinburgh and Glasgow) and three regions during 1974–2018. Increases in mortality risk under extreme cold and heat in individual cities and regions were aggregated using multivariate meta-analysis. Cold results are summarised by comparing the relative risk (RR) of death at the 1^st^ percentile of localised temperature distributions compared to the 10^th^ percentile, and heat effects as the RR at the 99^th^ compared to the 90^th^ percentile.

**Results:**

Adverse cold effects were observed in all cities and regions, and heat effects were apparent in all cities and regions. Aggregate all-cause mortality risk in Scotland was estimated to increase by 9% (95% confidence interval, CI: 8%, 11%) under extreme cold and 4% (CI: 3%, 5%) under extreme heat. The elderly had the highest RR under both extreme cold and heat. Males experienced greater cold effects than females, whereas the reverse was true with heat effects, particularly among the elderly. Those who were unmarried had higher RR than those married under extreme heat, and the effect remained after controlling for age. The younger population living in the most deprived areas experienced higher cold and heat effects than in less deprived areas. Deaths from respiratory diseases were most sensitive to both cold and heat exposures, although mortality risk for cardiovascular diseases was also heightened, particularly in the elderly. Cold effects were lower in the most recent 15 years, which may be linked to policies and actions in preventing the vulnerable population from cold impacts. No temporal trend was found with the heat effect.

**Conclusions:**

This study assesses mortality risk associated with extreme temperatures in Scotland and identifies those groups who would benefit most from targeted actions to reduce cold- and heat-related mortalities.

**Supplementary Information:**

The online version contains supplementary material available at 10.1186/s12940-022-00912-5.

## Introduction

In line with the UK hosting the 26^th^ United Nations Climate Change Conference of the Parties (COP26) in Glasgow, Scotland in 2021, there is growing recognition of the dangers heat stress poses to public health, even in higher latitude settings. Climate change is unmistakeably evident in Scotland, with average temperatures between 2011–2020 being 0.9 °C warmer than the 1961–1990 average [54]. The mean temperature in Scotland is projected to continue rising with heatwaves projected to become more frequent [71, 94], yet little is known about the impacts of high temperatures on human health in Scotland. Even though England has had a Heatwave Plan and Heat-Health Warning System in operation for almost 20 years now [85], there are still no such measures in the devolved countries of the UK, including Scotland. This may be based on the assumption that heat is not a public health risk factor in Scotland, but this topic has not been fully explored to date [18].

Reflecting this, the majority of research to date on temperature-related mortality risk for the UK has been conducted on populations in England, especially London [9, 13, 49, 53]. There are few studies from Scotland: a literature search in 2019 only yielded 8 potentially relevant journal articles (See search terms, exclusion criteria and literature list in Appendix 1) and of these, only one paper looked explicitly at cold effects on daily mortality in Scotland [22]. Another paper studying temperature thresholds corresponding to the lowest mortality risk in three European countries including Scotland was published recently [29].

Previous research found spatial heterogeneity in cold and heat effects between populations resulting from regional acclimatisation and varying distributions of vulnerability factors such as socioeconomic status [28, 47, 51, 53]. Evidence on temperature-mortality relationships in one city or region is therefore not readily applicable to other settings and hence is ill-equipped to support policies and interventions in other places [5]. The mortality rate in Scotland, especially in Glasgow, has been higher than in the rest of Britain and other western European countries since the 1950s with multiple contributory factors such as inequality, negative health behaviours, and lagged effects of high historical level of deprivation, overcrowding and poor economic, urban development and planning policies [60, 62, 95]. Therefore, Scotland may also be more vulnerable to ambient high and low temperatures compared to many countries despite its relatively moderate climate, and hence there is a need to characterise the temperature-health risk present in Scottish populations.

An effective heatwave plan or heat-health warning system needs to be able to identify and support the most vulnerable population groups during extreme weather, and so characterising risk based on demographic (e.g. age, gender), socioeconomic factors (e.g. deprivation) and other risk factors are essential [4, 13, 92, 93]. Among social factors, being unmarried and living alone have been found to increase the impact of extreme temperatures on mortality in some studies, but it was not clear whether this was independent of the effects of age [33, 76]. The most vulnerable members of society may be at higher risk of extreme temperatures, which can be further exacerbated by future climate change and contribute to climate injustice [10, 80].

In this paper, we address current gaps in knowledge of how low and high ambient temperatures affect mortality risk in Scotland and associated vulnerable subgroups using extended time series datasets based on daily mortality records for the past 45 years for Scotland, including the four largest cities (Aberdeen, Dundee, Edinburgh and Glasgow) and northern, western and eastern Scotland. The three main objectives of this study are to a) investigate the relationship between ambient daily temperature and mortality risk; b) explore trends in the cold/heat-related mortality risk over time; c) assess the variation of cold/heat-related mortality risk by demographic and socio-economic features including age-group, sex, marital status, area-level deprivation and cause of death.

## Materials and methods

### Data

#### Mortality

**Fig. 1:**
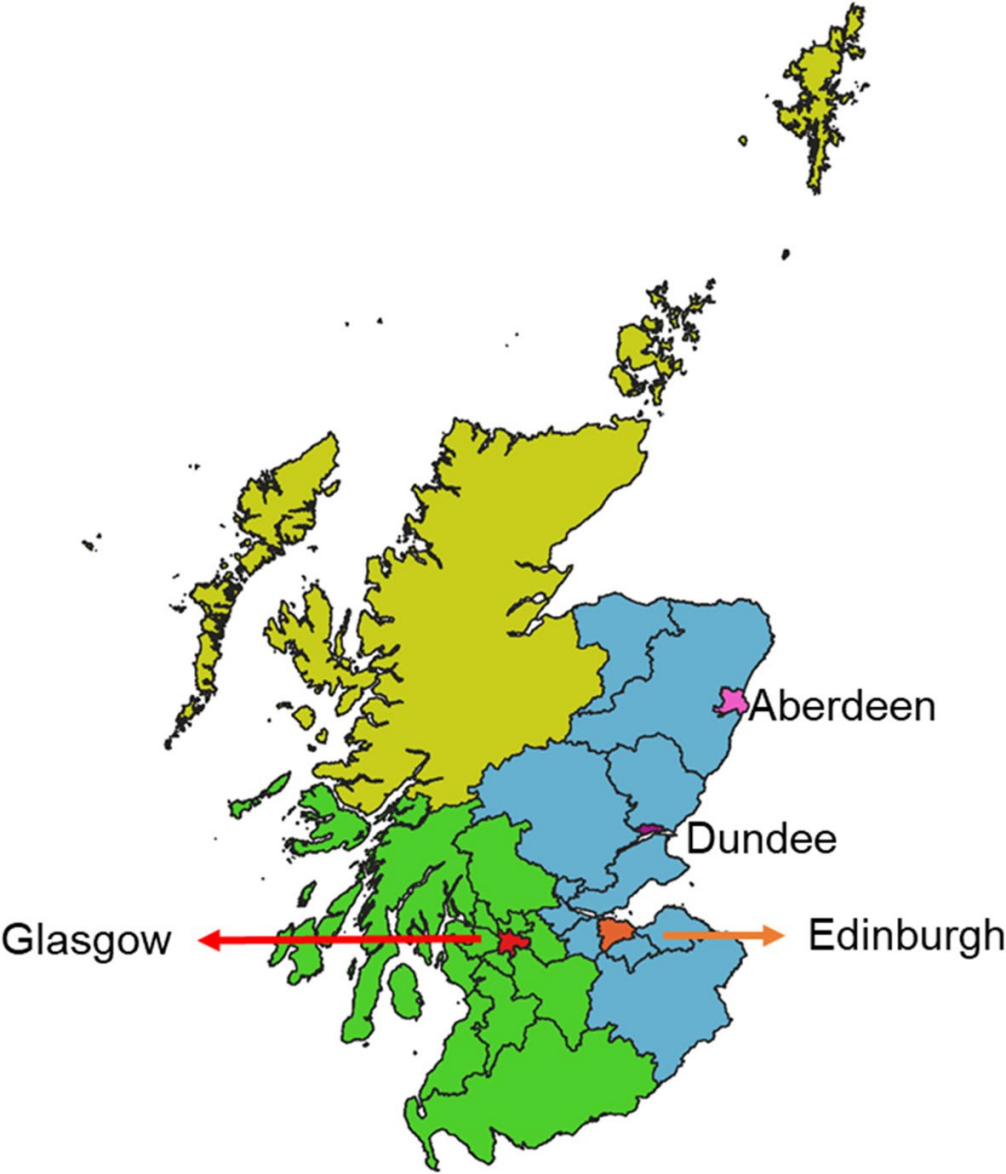
Study region: the four cities (as marked) and three regions (North—yellow, west—green and east—blue) in Scotland

Daily mortality counts in Scotland between 1974–2018 were obtained from National Records of Scotland. The daily mortality counts were aggregated into the four Scottish cities (i.e. Aberdeen, Dundee, Edinburgh and Glasgow) and three regions (East, West and North excluding the four cities). The location of the cities and regions are shown in Fig. 1, and the local authorities included in each region are listed in Appendix 2. The three regions were identified in accordance with the regions for which climate summaries are provided by the UK Met Office [64–66].

Attributes of the mortality data refer to the features of the deceased, including age group (0–64, 65–74, 75–84, 85 and over), sex, marital status (married, unmarried), underlying cause of death and socio-economic deprivation. Those who were unmarried include never married, divorced and widowed, and so can include both younger and older populations. Previous studies found that mortality from multiple diseases increased under ambient cold and heat, particularly cardiovascular and respiratory diseases [6, 7, 48]. Therefore, as well as all-cause mortality, separate assessments of these two main causes of death are also made. Table A2 lists the International Classification of Diseases (ICD) codes for underlying causes of death in different time periods.

Socioeconomic deprivation is represented by the Carstairs Index, which is originally constructed using Scottish Census outputs in 1981 on postcode sectors as a measure of material deprivation and has been widely used to study health inequality in Scotland [20, 23, 57]. Although the Scottish Index of Multiple Deprivation, developed by the Scottish Government, is a more up-to-date deprivation index, it is only available since 2004 and so cannot cover the whole study period [75]. The original Carstairs Index is composed of four indicators: overcrowding, unemployment among men, low social class and not having a car [24]. In this study, the Carstairs Index was modified by replacing the male unemployment component with total unemployment to reflect the increase in female participation in the labour market (e.g. the employment rate of Scottish females increased by 10% between 1992 and 2018) [79]. This is expected to have little effect on the Carstairs score because a pre-analysis showed high correlations between the percentage of male and total unemployment (0.96, 0.95, 0.92 and 0.91 in 1981, 1991, 2001 and 2011 respectively) (Figure A1). However, the decreasing correlation also indicates that male unemployment becomes less representative of the total unemployment situation in society over time. To calculate the modified Carstairs scores, the neighbourhood-level data for each component were extracted from the Scottish census outputs in 1981 (on enumeration area level), 1991, 2001 and 2011 (on census output area level). The census variables used for the calculation of the modified Carstairs Index are listed in Appendix 3.1. The modified Carstairs score for each small area was an unweighted combination of the four components that were standardised to have a mean of zero and variance of one. The spatial distribution of the deprivation quintiles is shown in Figure A2. Population weighted quintiles of modified Carstairs scores were derived and linked to daily mortality counts in the same small area for stratified analysis by deprivation level.

**Fig. 2:**
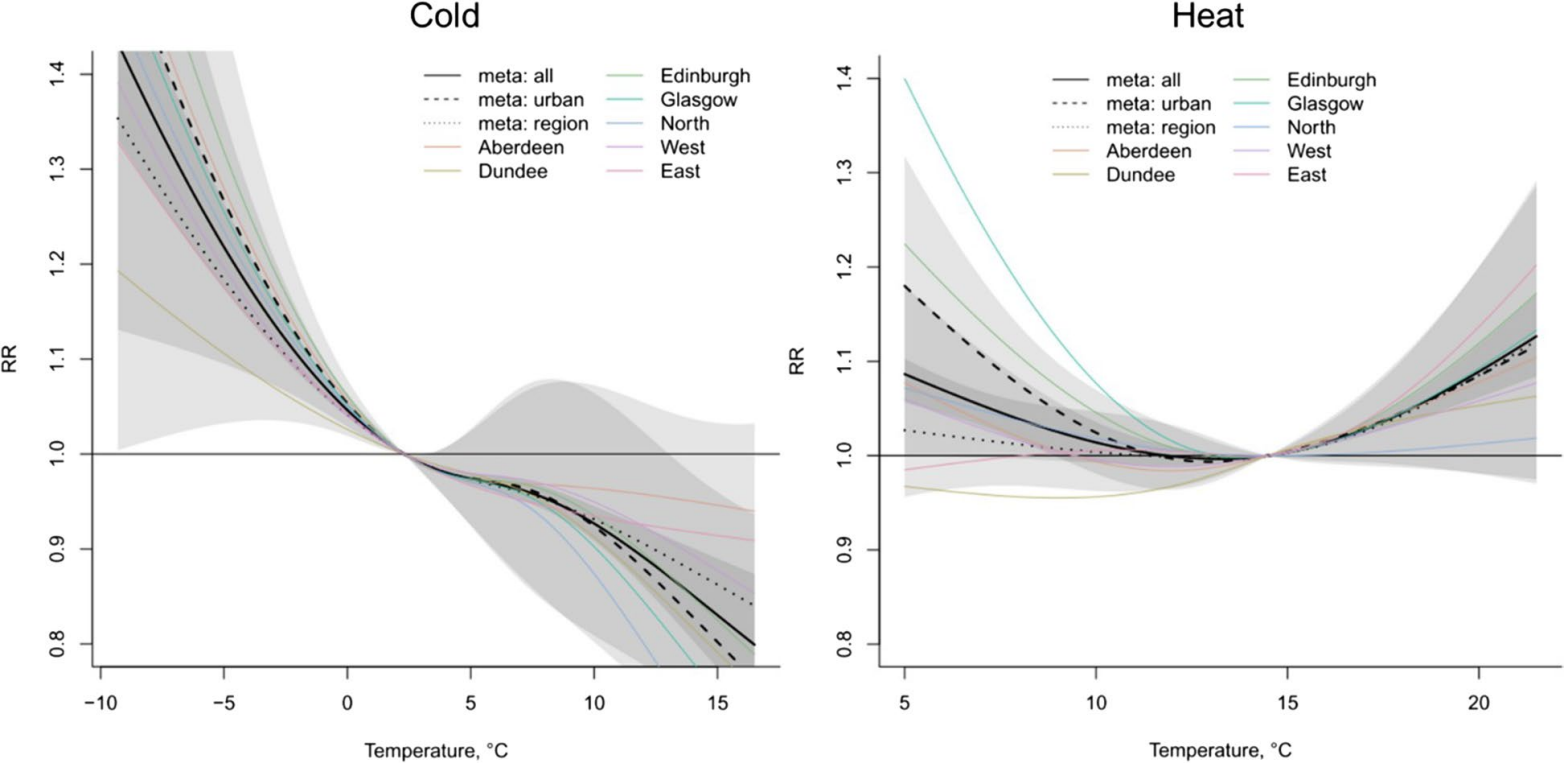
Relative risk under daily mean temperatures in each city and region and meta-analysis results in October to April (next year) for cold effects (left) and in June, July and August for heat effects (right) in 1974–2018

#### Temperature

Daily maximum and minimum ambient temperatures were obtained from HadUK-Grid Gridded Climate Observation (v1.0.0.0) [50] on 1 km grid and downloaded from the Centre for Environmental Data Analysis [67]. The data in each of the four cities and three regions were cropped with the digital boundaries of the local authorities obtained from EDINA Digimap Service (the data in the four cities were excluded from the three regions) and area-level mean daily maximum and minimum temperatures were calculated. The daily mean temperature of each locality was taken as the average of the area-level mean daily maximum and minimum temperature.

#### Air pollution and relative humidity

Other meteorological variables and air pollution may confound the relationship between temperature and mortality. Due to data limitations, only data on relative humidity, particulate matter with an aerodynamic diameter smaller than 10 µm (PM_10_), and ozone for Edinburgh during 2004–2018 were available for sensitivity analysis. The relative humidity data were monitored at Edinburgh Royal Botanical Garden and downloaded from MIDAS Open: UK hourly weather observation data. The air pollution data were monitored at Edinburgh St Leonards and downloaded from the Scottish Environmental Protection Agency.

Missing data of less than 20 consecutive days were imputed using a combination of seasonal decomposition and linear interpolation. Missing data with larger gaps were imputed with the mean relative humidity value at the Edinburgh Royal Botanical Garden and the mean PM_10_ and ozone concentration at Edinburgh St Leonards during 2004–2018 without the missing data. This was conducted using R package ImputeTS.

### Statistical analysis

Firstly, city- and region-specific time-series quasi-Poisson regression analysis with distributed lag models was performed. The outcome was daily mortality count and the predictor was daily mean temperature. Quasi-Poisson regression was used to account for potential overdispersion of the data [84].

The effect of low and high temperatures on increased mortality risk (cold and heat effect thereafter) may last over a period of time (i.e. lagged effect), with a longer lag for the cold effect (e.g. 14–28 days) and a short lag for the heat effect (0–3 days) found by previous studies [6, 38]. In this study, distributed lag models were used to take into account lagged effects [35]. The modelled result is the accumulated relative risk (RR) under the same day and lagged exposures on previous days. The analysis was done in R/RStudio using package dlnm [34].

A maximum lag of 14 days and 1 day were considered for the cold and heat effects respectively in this study. The analysis of cold and heat effects was conducted separately by using data in October through to April next year (OtA for short) and June, July and August (JJA) respectively. The transitional months of May and September were excluded from the analysis for either cold or heat effects. Some previous studies also separated the analysis by season or restricted the analysis of the heat effect to summer months and the cold effect to winter months [14, 56].

Previous studies found that when using a long lag (e.g. 4 weeks) for the heat effect, the all-cause mortality risk due to temperature increase is lower than using shorter lags in London, which may be due to mortality “displacement” where the mortality of those who are in advanced forms of illness is brought forward [44]. In addition, a simulation shows that when the lag of an exposure–response relationship is relatively short (e.g. 3 days), using a distributed lag structure of 7 days to model the relationship resulted in a larger root mean square error than using dummy variables for individual lag days over lag 0–3 [36]. Therefore, using separate models with appropriate lag times allows for capturing the maximum cumulative effect within the lagged period as well as reducing imprecision due to the inclusion of longer lags.

In OtA, the relationship between the lag days and daily mortality is modelled using a natural cubic spline (NS) with two internal knots equally-spaced on the log scale. In JJA, dummy variables at lag 0 and lag 1 were used to model the lag-response relationship.

The relationships between daily mean temperature and mortality in OtA/JJA were first explored using distributed lag non-linear models using a NS for temperature with internal knots at the 30^th^ and 70^th^ percentile of the daily mean temperature in OtA/JJA averaged for the four cities and three regions. Placing the knots at the same absolute temperature in all cities and regions allows for aggregating the relationships through meta-analysis easily, and it is expected to have little effect on the result compared to using different knots in different cities/regions as the temperature difference across the cities and regions is generally small and the result is relatively robust to the placement of knots (see the sensitivity analysis section and the result in Figure A4).

Long-term trends and medium-term variations over time may confound the relationship between daily temperature and mortality. For example, there is an overall long-term decrease in the number of deaths and mortality rate in Scotland since 1951, whereas the trend changed in 2012 with an increasing number of deaths and a stable mortality rate, particularly in the most deprived areas [77, 88]. This is likely affected by various factors, with austerity policies as the most likely causal factor including reduced public spending on services such as local government and healthcare, decreased incomes of the poorest (due to increased conditionality and real-terms value of social security payments) resulting in widening inequality [31, 61]. This long-term change and short-term variation in mortality count were controlled using an interaction term between indicators of year and a natural cubic spline of day-of-OtA with 5df for cold effects, and similarly an interaction term between year and a natural cubic spline of day-of-year with 3df for heat effects. Day-of-week and public holidays were also controlled using dummy variables.

Previous studies show that the heat effect usually starts to appear at a more extreme temperature than cold, with the temperature corresponding to the lowest mortality risk at roughly the 90^th^ percentile of the daily temperature distribution in London [38, 45]. The mechanisms under moderate and extreme cold may differ with direct cold effects more likely to occur under extreme cold [6]. In this study, we aim to study the impacts of extreme temperatures. Therefore, the RR is calculated by taking the ratio of the mortality risk under a temperature in relation to the mortality risk at the 10^th^ and 90^th^ percentile of annual temperature distribution averaged across the four cities and three regions for OtA and JJA respectively (corresponding to 2.3 °C and 14.5 °C).

The city- and region-specific results were then combined through multivariate meta-analysis using R package mixmeta [37]. In addition, a meta-regression of the results with indicators of urban and rural region as a meta-predictor was also conducted.

To explore the change in the cold and heat effect over time, the data were stratified into three 15-year periods (i.e. 1974–1988, 1989–2003, and 2004–2018). The dlnm models described above were applied to these time periods. Meta-regression of time period allowing for random effects across study regions was conducted to pool the results in individual cities and regions to obtain a national-level result.

We also performed subgroup analysis by demographic factors (age, gender and marital status), cause of death and small area-level deprivation indicated by the Carstairs Index (Mortality section) by refitting the above-described dlnm stratified by the subgroups. The results in individual cities and regions were combined using meta-regression with indicators of subgroups while allowing for random effects across study regions for a national result. Age may confound the effect of gender, marital status and deprivation on the heat and cold effect. For example, a higher mortality risk among females may be confounded by age because a higher proportion of the older population is females due to their longer life expectancy [73]. Therefore, we performed further analysis by stratifying the mortality data by subgroups crossing age and sex, marital status and deprivation. To increase the number of observations in each subgroup, the age groups were reduced into two categories: age 0–74 and 75 and above representing the younger and older populations. For example, there were four subgroups resulting from our age and sex categorisation: young females, young males, old females and old males. The city- and region-specific subgroup results were also combined using meta-regression with indicators of subgroups and random effects across study regions to represent a national result.

The increase in mortality risk under extreme temperatures was summarised with the RR at the 1^st^ and 99^th^ percentile of the annual temperature distribution averaged across the four cities and three regions (corresponding to -1.7 °C and 17.9 °C) and the associated 95% confidence intervals (CI) for the cold and heat effects respectively.

### Sensitivity analysis

Multiple analyses were undertaken to explore the sensitivity of the main model to certain methodological choices. We conducted sensitivity analyses with varying lengths of the lag period, different number and placements of the knots of the temperature NS and different methods for controlling the long-term trend and variation in time. Relative humidity and air pollution including ozone and PM_10_ in Edinburgh between 2004–2018 were also included in the model as a sensitivity analysis. Table A3 lists the sensitivity analyses carried out and the difference in the model settings with the main model.

## Results

The daily mean temperature and daily median mortality count in each city and region are summarised in Table 1. The average daily mean temperature across the four cities and three regions is 5.4 °C in OtA and 13.6 °C in JJA. Daily mean temperatures are higher in the four cities than in the three regions (Table 1).

**Table 1:** Average daily mean temperature and median mortality in each city and region in the whole year, October to April (next year) (OtA) and June, July and August (JJA) between 1974–2018

	Daily mean temperature			Daily median mortality		
Region	Whole year	OtA	JJA	Whole year	OtA	JJA
Aberdeen	8.3	5.4	13.4	6	7	6
Dundee	8.8	5.7	14.4	5	5	5
Edinburgh	8.7	5.6	14.1	14	14	12
Glasgow	9.2	6.0	14.7	23	24	21
North	7.8	5.3	12.1	9	9	8
West	8.2	5.1	13.4	58	62	53
East	7.8	4.7	13.2	45	48	41

The daily median mortality count in the whole year ranges from 5 in Dundee and 6 in Aberdeen to 45 and 58 in eastern and western Scotland respectively (Table 1). Median mortality count is generally larger in OtA than in JJA (also see Table A4 for daily temperature and mortality count in each month).

Comparing the mortality count among subgroups (Table A5), there is a higher proportion of mortality count among those who are 75–84 years’ old than other age groups, slightly more females than males except in the North, and more unmarried than married. A large proportion of mortality is due to cardiovascular diseases and other causes, with less due to respiratory diseases (Table A5). In Dundee, Edinburgh, Glasgow and Western Scotland, there is more mortality in the most deprived quintile than the least deprived quintile; while it is reversed in Aberdeen, North and East (Table A5).

There are also interaction effects of the mortality attributes (see total mortality count by subgroup interactions in Table A6). Among the mortality at younger ages (age 0–64 and 65–74), there are more males than females, and more married than unmarried; whereas it is reversed among the older age groups (75–84 and 85 and above). More deceased females are unmarried than married, whereas more males are married. Among the five quintiles of deprivation, there is a higher proportion of mortalities in the youngest age group (0–64 years’ old), males and unmarried who are from the most deprived neighbourhood compared to their counterparts.

The city- and region-specific and meta-analysis results of the relationships between RR and daily mean temperature in OtA and JJA are shown in Fig. 2. The meta-estimation of RR in all cities and regions at the 1^st^ percentile compared to the 10^th^ percentile of the annual temperature distribution is 1.09 (CI: 1.08, 1.11) for the cold effect, and the meta-estimation of RR at the 99^th^ percentile compared to the 90^th^ percentile of daily temperature distribution is 1.04 for the heat effect (CI: 1.03, 1.05) (Table A7).

For the cold effect in OtA, as the temperature decreases, the RR increases non-linearly in all cities and regions (Fig. 2). The meta-analysis results show that as the temperature decreases in OtA, the RR increases rapidly when the temperature is below 3 °C or between 9 °C and 15 °C, and there is a levelling-off period in-between.

In JJA, there is a heat effect in all cities and regions (Fig. 2). The meta-analysis results show an evident increase in mortality risk as the temperature increases when the temperature is above 14.5 °C.

**Fig. 3:**
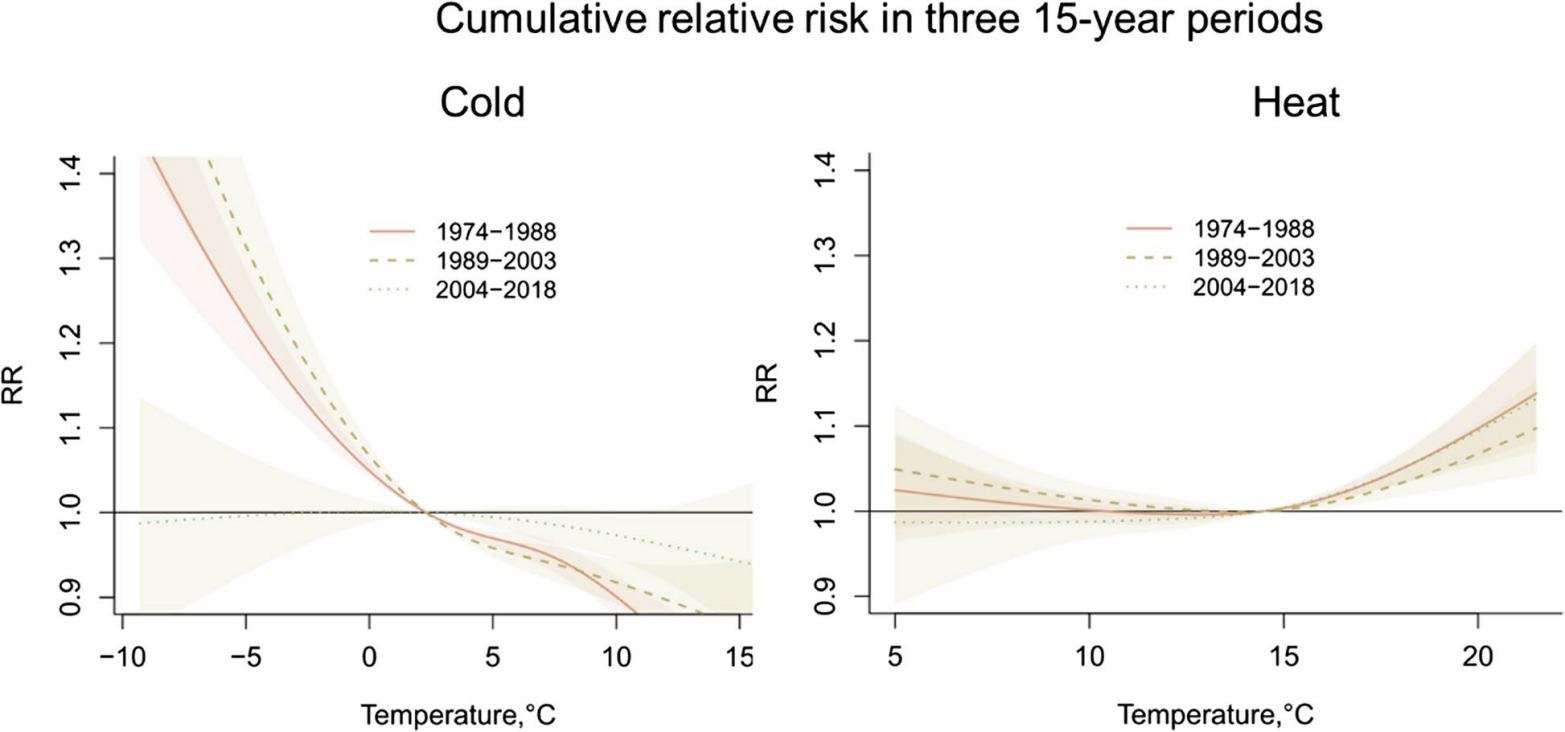
Meta-estimation of cumulative relative risk under daily mean temperatures in Scotland in October to April (next year) for cold effects (left) and in June, July and August for heat effects (right) in three 15-year periods

The cold and heat effects in each of the three 15-year periods are illustrated in Fig. 3. The cold effect is vastly reduced in the most recent 15 years. In contrast, there is generally less temporal variation in the heat effect (Fig. 3).

**Fig. 4:**
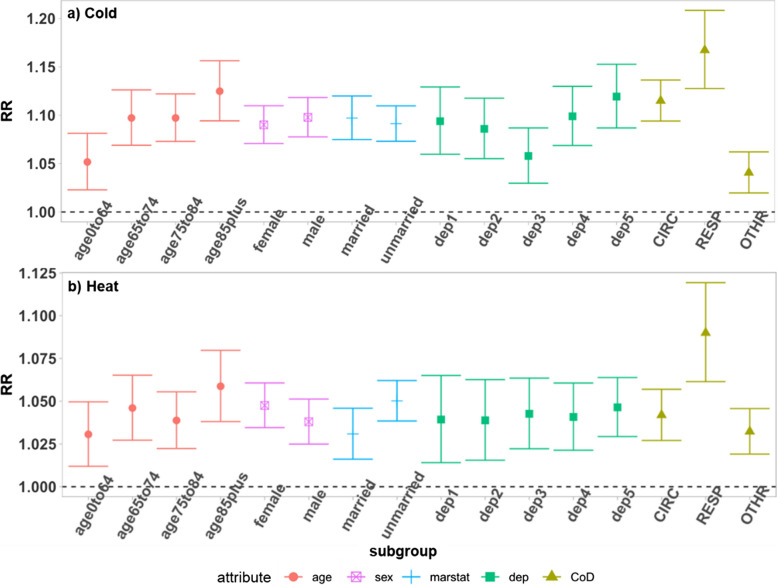
Meta-estimation of RR of subgroups at (**a**) 1^st^ daily temperature distribution for cold effects and (**b**) 99^th^ daily temperature distribution for heat effects. The full terms for the abbreviations are: marstat: marital status,,dep: deprivation, dep1-5: quantile 1–5 of deprivation (from the least deprived to the most deprived), CoD: cause of death, CIRC: cardiovascular diseases, RESP: respiratory diseases, OTHR: causes of death other than CIRC and RESP

The meta-estimation results of the RR of subgroups at the 1^st^ and 99^th^ percentiles for the cold and heat effect are shown in Fig. 4 (also see values in Table A8). There is an increase in the mortality risk among all age groups under extreme cold, and the risk is higher for the older age groups (Fig. 4a). For the heat effect, the relative risk is the highest in the oldest age group (Fig. 4b). The result also shows that males have a slightly higher cold-related mortality risk than females, while females have a slightly higher heat-related mortality risk than males (Fig. 4).

Extreme cold increases the mortality risk of both those who are married or unmarried, and for the heat effect, those who are unmarried experience a higher risk than those married (Fig. 4). Those in the most and least deprived area experience slightly higher cold effects compared to other deprivation quintiles (Fig. 4).

During exposure to cold and heat, there is an increase in mortality risk from all causes under investigation, with the highest increase in risks for respiratory deaths (Fig. 4).

**Fig. 5:**
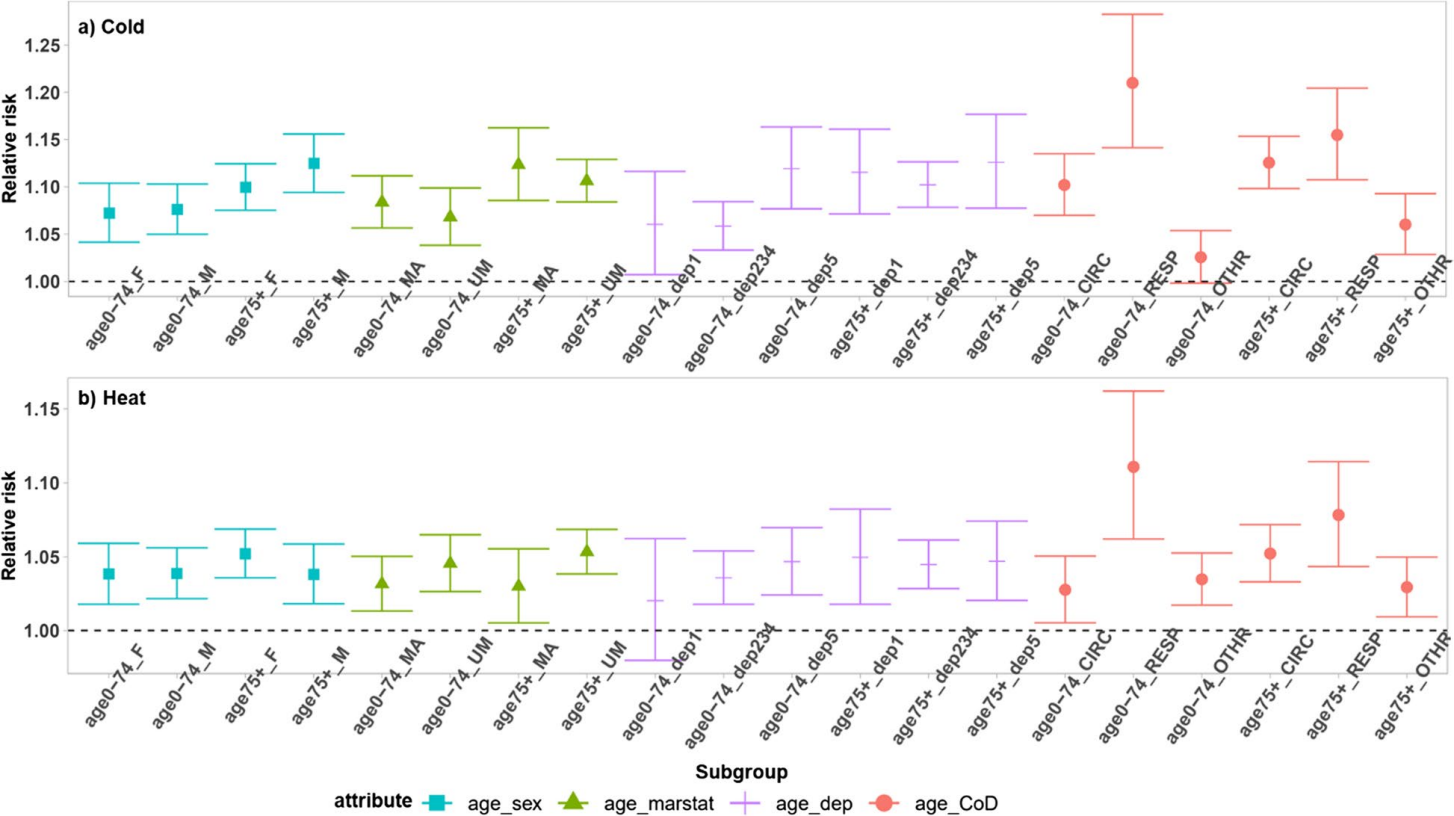
Meta-estimation of relative risk of subgroups (**a**) at the 1st percentile of temperature distribution compared to the 10th percentile for cold effects, and (**b**) at the 99th percentile of temperature distribution compared to the 90th percentile for heat effects. The full terms for the abbreviations are: female (F), male (M), married (MA), unmarried (UM) (See the full term of other abbreviations in the caption of Fig. 4). The underline joins two attributes, e.g. age0-74_F represents those who are at the age of 0–74 and also being female

The cold and heat effects by sex, marital status, deprivation and cause of death when controlling for age are shown in Fig. 5 (also see values in Table A8). This shows that, after controlling for age, the higher cold-related mortality risk of males than females and the slightly higher heat-related mortality risk of females are more evident among the older age group. The married population experience a slightly higher cold-related mortality risk than the unmarried population, whereas those who are unmarried experience a slightly higher heat-related mortality risk regardless of age. Among the younger age group, those in the most deprived areas have higher cold and heat effects. The mortality risk from cardiovascular diseases is higher among the older population than the younger population under both extreme cold and heat, whereas the mortality risk from respiratory diseases is higher among the younger than the older population.

The overall pattern of the temperature-mortality relationships from the sensitive analyses does not change compared to the main model as introduced in Statistical analysis section (Figures A3, A4, A5 and A6).

## Discussion

This study found increased mortality risk under both low and high temperatures in Scotland. There was a continuous but non-linear increase in the mortality risk as the temperature decreased in OtA. In JJA, mortality risk started increasing as the temperatures increase above around 14.5 °C in Scotland. This is comparable to the temperature thresholds corresponding to the lowest mortality risk (heat threshold thereafter) in Scotland found in another study [29] but lower than previous studies in other places [13, 38, 39]. This indicates that the heat-health impacts can also be observed at relatively low temperatures in places with a cool climate. It may partly be because of the acclimatisation and adaptation effect where people and society are adapted to their local climate as previous studies found higher heat thresholds in warmer places than cooler places of Europe, e.g. around 30 °C in Athens and Rome and around 20 °C in Helsinki and Stockholm [13, 38, 39].

Due to a historically cool climate, the cultural, behavioural and policy focus in Scotland has been the reduction of cold impacts with heat largely remaining an invisible risk, which is further discussed below. People may experience enhanced heat exposure indoors as the focus of building design and energy efficiency has been to keep buildings warm [43, 82], which may contribute to the low heat threshold in Scotland as well. It raises research and policy needs in considering city/region/country-specific conditions for the estimation of cold/heat-health burdens and the design of cold/heat-health warnings and strategies. Although some of the mortalities under heat may have been brought forward from those in advanced forms of illness, a significantly elevated heat risk was observed in all age groups in our study. Our results are useful in indicating the acute increase in mortality risk for health services to be more prepared for increased needs in health and emergency services during hot weather.

The RR at the 1^st^ percentile compared to the 10^th^ percentile of daily temperature distributions (i.e. 1.7 °C and 2.3 °C) was 1.09 (CI: 1.08, 1.11) to represent the cold effect, and the RR at the 99th percentile compared to the 90th percentile of temperature distribution (i.e. 17.9 °C and 14.5 °C) was 1.04 (CI: 1.03, 1.05) for the heat effect. In line with previous studies, the heat effect in cities was found to be higher than in rural regions [48].

The cold effect in Scotland was comparable to England where a 3.44% (CI: 3.01, 3.87) increase in all-cause mortality for each degree decrease at low temperatures was found [46]. However, caution is needed in making direct comparisons because of differences in thresholds, lag structures, and other model specifications [6]. The heat effect in Scotland was smaller than in England and Wales as a whole, where a mean increase of 3% (CI: 2%-3%) in mortality risk for each degree increase under high temperature was found [48]. The heat effect in Scotland was more comparable to that found in North East England and Wales, where there was an increase of 1.7%-2.0% for each degree increase in high temperature [48].

Increased mortality risk under extreme cold was found to have decreased hugely to a low level in the most recent 15 years, which may be because cold-health impacts have been widely identified, for example, as a consequence of fuel poverty [15, 16, 89], and hence policies and actions have been developed to mitigate the impacts on vulnerable populations. These policies include the Cold Weather Payments launched in 1988 [90], the Winter Fuel Payment scheme that was introduced in 1997 and the substitute Winter Heating Assistance since 2016, and the Warmer Homes Scotland scheme which provides support for insulation, efficient heating and renewable technologies since 2015 [97]. These actions provide support to those who are most vulnerable to cold effects, e.g. pensioners (i.e. the elderly) and benefits recipients (e.g. low income, unemployment, disability).

Some policies like the Winter Fuel Payment and Cold Weather Payments were introduced UK-wide, and may explain a decrease in cold effects in England and Wales also during 1976–2005, although a few years had typically high cold effects, e.g. in 1976, 1986, 1989, 1997 and 1999 [25]. By contrast, there has been little awareness of the heat-health impacts in Scotland [1], which leaves heatwaves as an invisible risk in public health [18]. This potentially explains the little change in the heat-related mortality risk in the past 45 years, indicating a need for interventions to prevent heat-health impacts, particularly among vulnerable populations, as well as to reduce health inequalities.

The oldest age group was found to experience the highest cold and heat effects, which is in line with most other studies [4, 26, 41, 46, 86, 92]. The older population had a higher RR in cardiovascular mortality than the younger group, which has also been found in previous studies [22, 40, 52]; in contrast, the younger group had a higher cold-related mortality risk in respiratory diseases than the older group. Although less common, a higher cold-related respiratory mortality risk among the younger population was also found in Chicago, USA [78] and Spain [3]. This may be related to the higher prevalence and incidence of asthma among children, young and middle-aged adults [19], and a higher proportion of smokers among the younger population [11], or the elderly tend to stay indoors when the outdoor temperature is low and hence avoid the exposure to cold and infectious diseases [3].

Males were found to experience higher cold-related mortality risk, which remained after controlling for age. This may be associated with physiological, lifestyle and behavioural factors, e.g. less likely to seek help from doctors [96] and to wear appropriate clothing and hats and gloves in cold outdoors [53], and also more likely to smoke [11, 91]. A higher cold-related respiratory mortality risk among males than females was found in Spain [2], but in a study in eight regions in Europe, females showed higher risks than males [53]. Therefore, more research is needed to investigate whether vulnerabilities by gender are region-specific.

Females experienced slightly higher heat-related mortality risk, which may partly be confounded by age due to their longer life expectancies. However, females may still be more vulnerable to heat as shown by the higher heat-related mortality risk among older females than older males in this study. Some possible explanations include physiological features such as a lower sweating ability [63], and menopausal effects such as elevated body temperature and sweating [8]. There are more older females living alone than older males, and females have been experiencing lower socioeconomic status than males in the past, e.g. having lower employment rate, lower payment and less representation in senior positions, which may contribute to their vulnerabilities [17, 72, 74].

Marital status as a risk factor is rarely investigated in previous research compared to age and gender. In this study, those who were unmarried had higher RR under extreme heat, which is still evident after controlling for age. The high vulnerability among the unmarried was also found in some previous studies, e.g. in France [33], Italy [30, 93] and the US [42]. All of the various unmarried states (being single, never married, being separated/divorced and being widowed) were found to be associated with elevated mortality risks, particularly relating to cardiovascular diseases in a cohort study with around 14,000 Scottish men and women [68]. Experiencing existing health conditions, physiological stresses and being more likely to be living alone and socially isolated may contribute to the vulnerability of the unmarried population under heat exposure [27, 33, 68]. Although living alone cannot fully explain the worse health status among the unmarried population, it has been identified as a significant risk factor for elevated mortality during a 1999 heatwave in Chicago, US [76].

Deprivation is usually considered to contribute to health inequalities due to a lack of material and social resources [51, 69]. Deprivation is differentiated from poverty in that deprivation can reflect multiple disadvantages, such as clothing, housing, household facilities, education, environmental, working and social conditions [83]. Those who are deprived may have fewer resources to prepare, respond and adapt to cold and heat [58]. This study found higher cold- and heat-related mortality risks among younger people who lived in the most deprived areas compared to younger people in less deprived areas, whereas no evident pattern was observed in the older population. Affluent old people may be more likely to live in big and old houses with lower energy efficiency, and are more likely to experience cooler indoor temperatures and a higher cold-health risk. This could be supported by the generally positive relationship between energy efficiency and deprivation in Scotland (Figure A). Further research on the modification effects of deprivation, particularly with the interaction of age on the cold and heat effects is needed.

Some previous studies found an increase in cold- and heat-related mortality risks among people in more deprived areas in England and Wales [39, 70]. Higher excess winter mortality was also found in regions with higher deprivation in Scotland [51]. However, evidence of the effect of deprivation on cold-/heat-related mortality is mixed and little effect of deprivation was found in two studies in the UK [12, 59] and most studies of the 2003 heatwave in Europe [55]. This may be because current deprivation indices cannot fully reflect socio-economic vulnerability to cold and heat exposures [58]. In addition, deprivation includes diverse dimensions which also vary over time and hence it is unlikely that any indicator could fully capture it fully [87]. For example, the difference in the age and gender standardised all-cause mortality rate after the adjustment of deprivation using the Carstairs Index between Scotland and England increased from 4 to 10% between 1981 and 2011 [87]. The decreased relevance of the Carstairs Index to health is, at least partially, due to changes in peoples’ lived experience and the relative importance of different aspects of deprivation [87]. Deprivation is often studied at small area levels, whereas caution is also needed when drawing inferences at an individual level [32, 81].

This study provides new evidence of the impacts of short-term exposure to extreme cold and heat on mortality in Scotland and associated vulnerable subgroups. It uses particularly long time-series of daily temperature and mortality for the four most populated Scottish cities and three regions in the past 45 years, which enables enough statistical power in the estimation of the mortality risk under extreme cold and heat. This study is novel in that, in addition to the investigation of the widely studied individual modifying factors such as age and sex, it also investigated the effect of marital status and socio-economic deprivation on cold- and heat-related mortality risk whilst controlling for age. The long time-series also allows the estimation of changes in cold and heat effects during multiple time periods and the assessment of long-term trends in heat and cold risk.

There are potential limitations in this study. Although seasonal variation in mortality has been controlled for, this study did not explicitly control for influenza epidemics which may leave residual confounding. However, the role of a factor being a confounder or effect modifier depends on its position on the causal pathway between temperature and mortality [21]. As temperature has direct impacts on influenza transmission, it is debatable whether influenza epidemics should be controlled as a confounder [6]. Due to the use of extreme thresholds, the number of observations beyond the thresholds is small, yielding limited statistical power in the stratified analysis. Therefore, formal tests of interaction effects were not assessed, and hence this paper focuses more on effect sizes rather than statistical significance. An averaged temperature series over a city or region is used in the study which may not reflect temperatures that individuals are exposed to at any time since individuals spend a large proportion of time indoors with different heating and ventilating situations. As a final limitation, marital status was only separated into married and unmarried, whereas the effect of cohabitation status and different subcategories of being unmarried, e.g. single, divorced, widowed could not be investigated.

## Conclusion

This is the first study exploring the temperature-mortality association for the whole population of Scotland and found increased mortality risk associated with both cold and heat exposure. Our results reveal that cold-related mortality risk has decreased markedly over time, with a minimal risk under extreme cold in the most recent 15 years, whereas there has been little temporal change in the heat effect. This indicates that heat-health risk in Scotland has remained an overlooked policy area. Since heat has been identified as a risk factor, at least in some vulnerable groups in Scotland, including the elderly, females, unmarried and people who have pre-existing conditions, particularly respiratory and cardiovascular diseases, further research should be conducted to identify modifiable factors that heighten heat-risk in these groups and also likely future health burdens under climate change scenarios. Such results could support heat-health policies and actions to prevent excess mortality during high temperatures in Scotland.

### Supplementary Information

**Additional file 1. **

## Data Availability

The mortality dataset analysed during the current study are not publicly available because the authors are not the creator of the dataset, but it is available from the National Records of Scotland on reasonable request. The temperature dataset of HadUK-Grid Gridded Climate Observation analysed during the current study can be obtained from Centre for Environmental Data Analysis https://catalogue.ceda.ac.uk/uuid/786b3ce6be54468496a3e11ce2f2669c.

## Abbreviations

GCCR: German Childhood Cancer Registry
C: Cardiovascular diseases
CI: Confidence interval
CoD: Cause of death
F: Female
ICD: International Classification of Diseases
JJA: June, July and August
M: Male
MA: Married
Marstat: Marital status
NS: Natural cubic spline
OtA: October to April next year
PM10: Particulate matter with an aerodynamic diameter smaller than 10 µm
R: Respiratory diseases
RR: Relative risk
UM: Unmarried
