# Correction: Temperature‑related mortality and associated vulnerabilities: evidence from Scotland using extended time‑series datasets

**DOI:** 10.1186/s12940-023-01011-9

**Published:** 2023-09-30

**Authors:** Kai Wan, Zhiqiang Feng, Shakoor Hajat, Ruth M. Doherty

**Affiliations:** 1 School of GeoSciences, University of Edinburgh, Edinburgh, UK; 2 Scottish Centre for Administrative Data Research, School of Geosciences, University of Edinburgh, Edinburgh, UK; 3 Department of Public Health, Environments and Society, London School of Hygiene & Tropical Medicine, London, UK; 4 Centre On Climate Change and Planetary Health, London School of Hygiene & Tropical Medicine, London, UK

**Correction: Environ Health 21, 99 (2022)**

**https://doi.org/10.1186/s12940-022-00912-5**

Following the publication of the original article [1], a mistake was found in the temperature data for the three regions in Scotland. This error affected some results marginally, however the conclusions and key messages remain valid after the error was corrected.

The daily mean temperature data was mislabelled during the analysis—the temperature data in the West was mislabelled as North, the temperature data in the East was mislabelled as West and the temperature data in the North was mislabelled as East.

All changes after and before the correction are presented Table 1. The section of the content and the line number of the updated manuscript are also included.

**Table 1:**
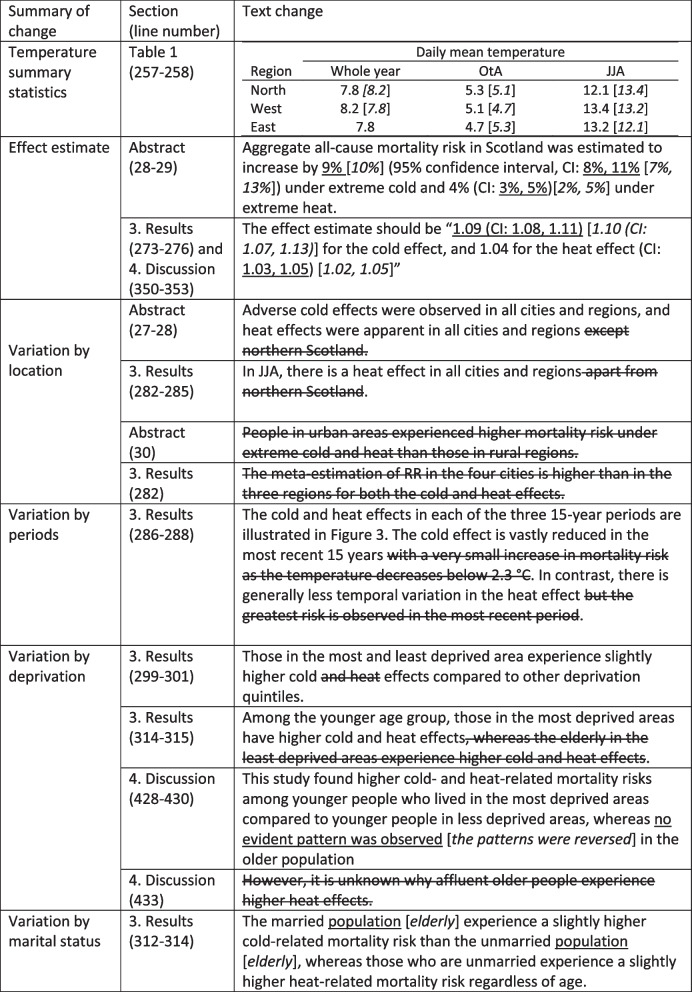
List of changes in text. Updated texts are underlined, and old texts are placed in square brackets in italic. The text deleted are marked with strikethrough

Figures 2, 3, 4 and 5 are updated, and the old and new figures are presented below.

**Fig. 2:**
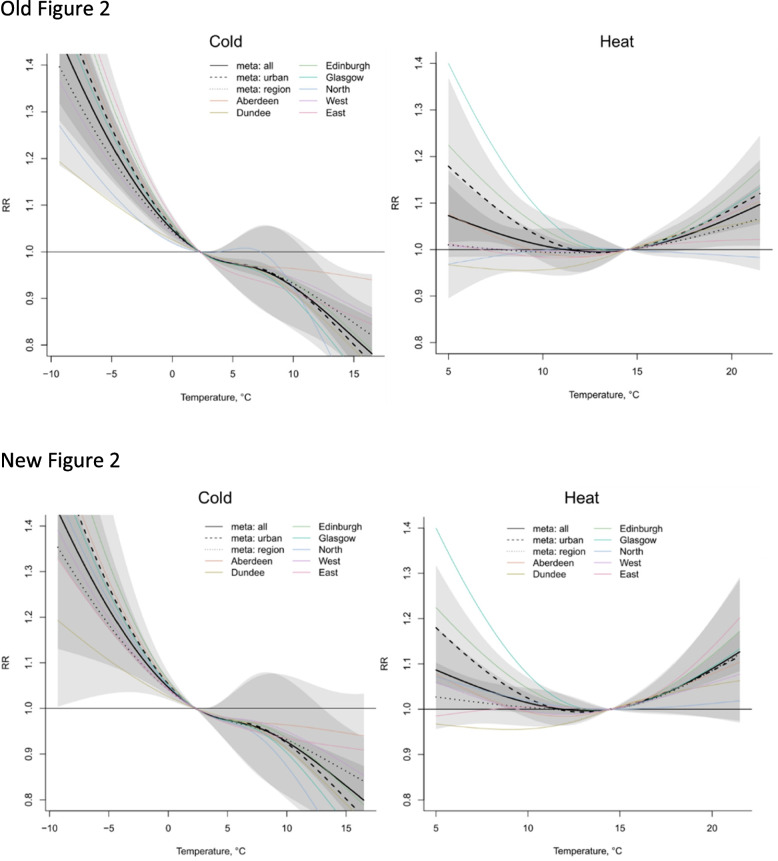
Relative risk under daily mean temperatures in each city and region and meta-analysis results

**Fig. 3:**
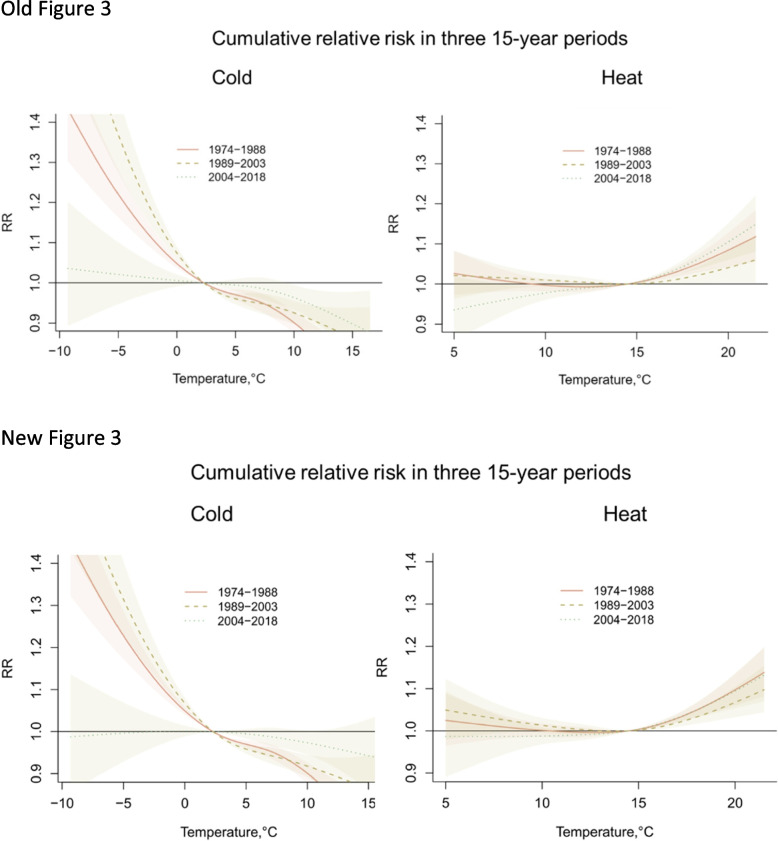
Meta-estimation of cumulative relative risk in three 15-year periods

**Fig. 4:**
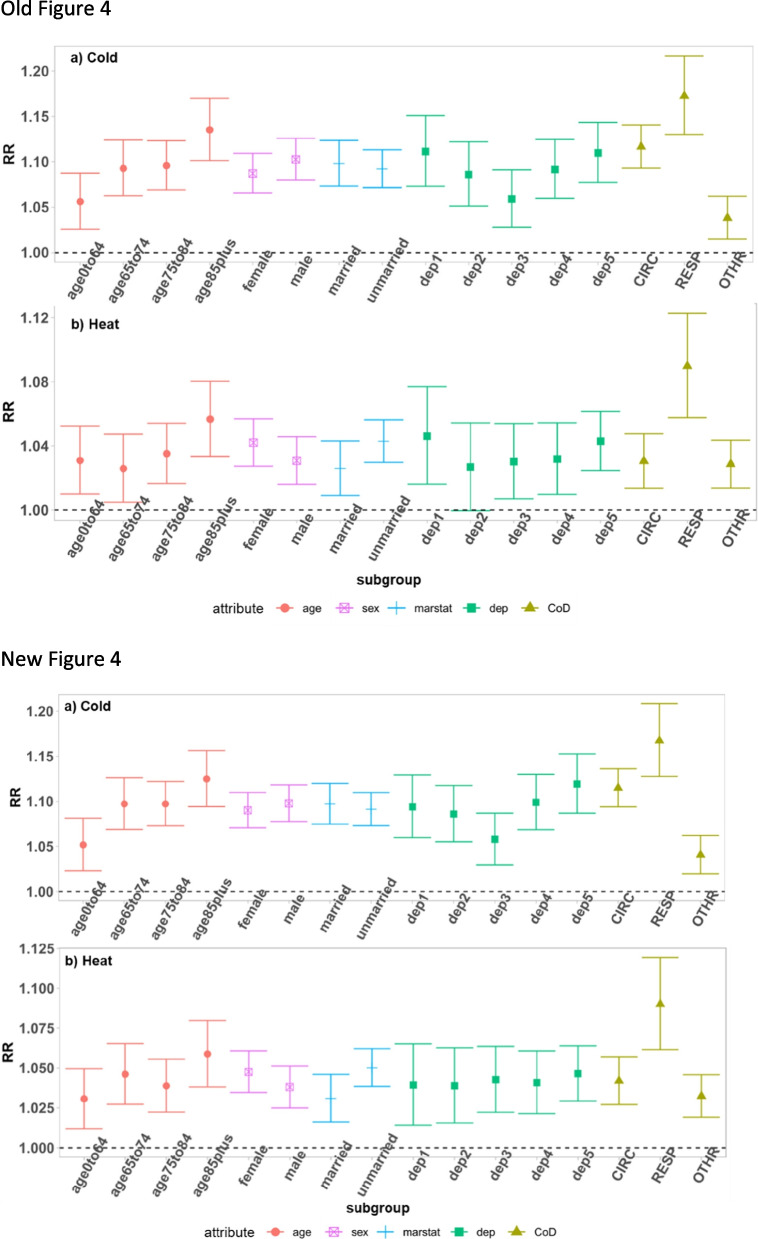
Meta-estimation of RR of subgroups

**Fig. 5:**
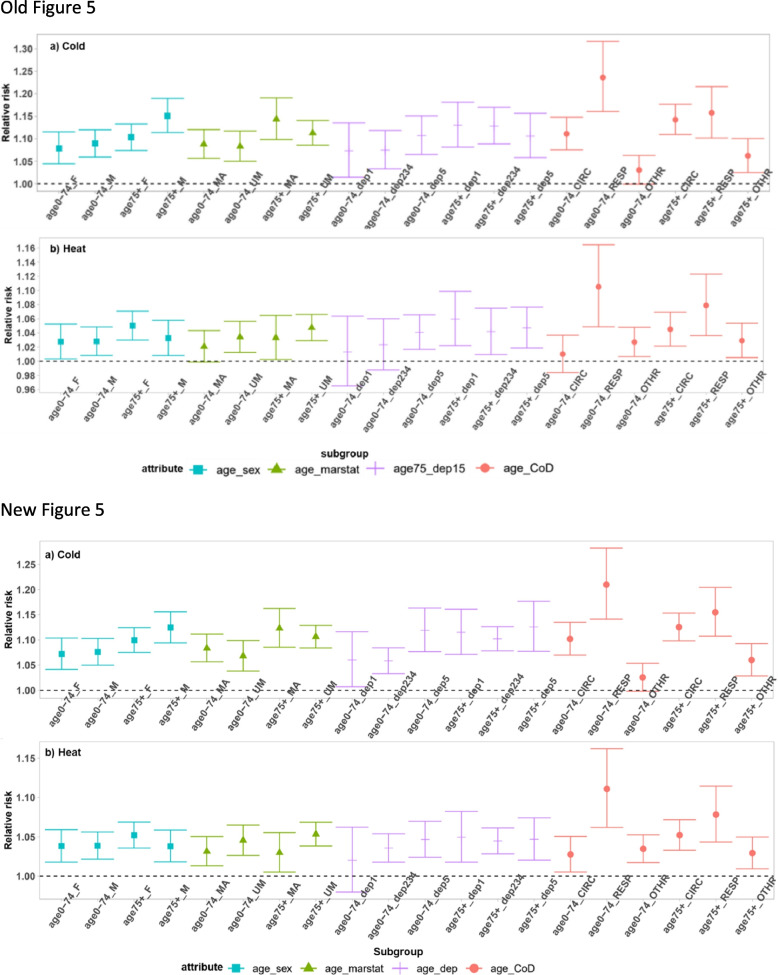
Meta-estimation of relative risk of subgroup interactions
