# Supplementary material for: Temperature-related mortality and associated vulnerabilities: evidence from Scotland using extended time-series datasets
Source: Environ Health. 2022 Oct 25;21:99. doi: 10.1186/s12940-022-00912-5 (PMC9594922; doi:10.1186/s12940-022-00912-5)
Supplement: Supplementary file 1 — Additional file 1. [file 12940_2022_912_MOESM1_ESM.docx]

# Appendix.

# 1. Literature search about temperature-related mortality in Scotland

Searching words: “temperature” and “mortality” and “Scotland”

Database: Web of Science Core Database

Date: 7^th^ November 2019

Results: 82 papers

Excluding by category: MARINE FRESHWATER BIOLOGY (27), FISHERIES (26), ECOLOGY (9), OCEANOGRAPHY (7), VETERINARY SCIENCES (5), ZOOLOGY (5), BIODIVERSITY CONSERVATION (2), FORESTRY (2), IMMUNOLOGY (2), INFECTIOUS DISEASES (2), ORNITHOLOGY (2), TOXICOLOGY (2), VIROLOGY (2), AGRICULTURE DAIRY ANIMAL SCIENCE (1), AIR POLLUTION (2), ANATOMY MORPHOLOGY (1), BIOTECHNOLOGY APPLIED MICROBIOLOGY (1), BODY TEMPERATURE (1), COMPUTER SCIENCE INTERDISCIPLINARY APPLICATIONS (1), GEOGRAPHY PHYSICAL (1), INTERNATIONAL RELATIONS (1), MICROBIOLOGY (1), OPERATIONS RESEARCH MANAGEMENT SCIENCE (1), SURGERY (1)

Further excluding studies that is not about Scotland: Hong Kong (1), Australia and US (1), Ireland (1), Quebec (1), UK or GB wide paper (2) and 1 study about Dolphin, 1 study about air pollution and mortality, and 1 study about blood pressure and room temperature instead of ambient temperature.

After refinement: 7 papers

Table A 1. Seven searching results about temperature-related mortality in Scotland

| Title | Study period | Study region | Health outcome | Time resolution | Method | Result |
| --- | --- | --- | --- | --- | --- | --- |
| Carder, et al. (2005) The lagged effect of cold temperature and wind chill on cardiorespiratory mortality in Scotland. *Occupational and Environmental Medicine*, 62(10), 702-710. | 1981-2001 (20y) | Edinburgh, Glasgow and Aberdeen | Mortality | Daily | Generalised linear Poisson regression with natural cubic splines to capture seasonal and other long-term time trend.  Lag days: 0, 1-6, 7-12, 13-18, 19-24, 25-30.  Software: Splus | Non-linear association between mortality and temperature.  The increase in mortality is steeper at temperatures below 11C.  When temperature is below 11C, a 1C decrease in daytime mean temperature is associated with an increase in mortality of 2.9% (all cause), 3.4% (cardiovascular), and 4.8% (respiratory).  “Wind chill” temperature was not found to be a better mortality prediction compared to “Dry bulb” temperature.  No significant variation among cities were found. |
| Dawson et al. (2008) Associations between meteorological variables and acute stroke hospital admissions in the west of Scotland. *Acta Neurologica Scandinavica*, 117(2), 85-89. | 1990-2005 (15y) | Glasgow | Morbidity (stroke) | Daily | Daily numbers of hospital stroke admissions were modelled using negative binomial regression. In some models of rare stroke events, where the negative binomial regression failed to converge, Poisson regression was used.  Software: SAS | Every 1C increase in mean temperature during the preceding 24h was associated with a 2.1% increase in ischaemic stroke admissions.  Higher maximum daily temperature gave a greater increase in lacunar stroke admissions. |
| Douglas et al. (1991) Seasonality of disease in Kuwait. *Lancet*, 337(8754), 1393-1397. | 1974-1988 (14y) | Northeast Scotland (Grampian region) & Kuwait | Mortality | Seasonal | Cosinor analysis. Provided data fit a unimodal sinusoidal curve, the best fit of a cosine-function curve to annual data is then  calculated. The year is taken as 360 degrees and  the each month of the year is assigned an angular value. Multiple regression analysis is completed between monthly data and sin (t) and cos (t). | There is winter-peak seasonality in  Kuwait similar in amplitude to that in Grampian even winter climate is most comfortable in Grampian and least comfortable in Kuwait.  In Kuwait (1980-83), around 25% deaths were among infants, while it’s < 1 % in Grampian.  In Grampian, deaths among those over 75 years of age constitute 48% of deaths, while in Kuwait they account for 24% of deaths |
| Gemmell et al & Watt, G. C. M. (2000) Seasonal variation in mortality in Scotland. *International Journal of Epidemiology*, 29(2), 274-279. | 1981-1993 (12y) | Scotland | Mortality | Seasonal | Seasonal pattern was analysed using a Poisson regression analysis.  The relationship between weekly mortality and temperature was assessed with a time series regression model. This method involves modelling the weekly mortality in a Poisson regionssion analysis and assessing the significance of short-term changes in weekly average temperature, controlling long-term and seasonal trends in mortality, serial autocorrelation, over dispersion and influenza epidemics.  Lag time: up to 5 weeks  Software: Splus | A seasonal variation in weekly death rates with a difference of about 30% (38%: 1981-1983; 26%: 1991-1993) between a summer trough and a winter peak, which is principally associated with respiratory disease, cerebrovascular disease and coronary artery disease.  A 1C decrease in mean temperature was associated with a 1% increase in deaths one week later.  When the temperature is 10-14C, the increase in mortality related to the decrease in temperature was negligible.  A significant fall in mortality was found when the temperature is over 14C and 1C decrease in temperature was associated with 1% fall in mortality.  No difference was found in the direct effects temperature on mortality when the data was analysed separately according to social class and area-based deprivation categories and no difference was found among the three cities.  The lack of evidence of a relationship between socioeconomic status and seasonal mortality may be due to that area-based measures of deprivation and individual-based social class data are not adequate proxy measures of housing conditions and fuel poverty. |
| Stewart et al. (2002) Heart failure in a cold climate - Seasonal variation in heart failure-related morbidity and mortality. *Journal of the American College of Cardiology*, 39(5), 760-766. | 1990-1996 (6y) | Scotland | mortality and morbidity (from heart failure) | Seasonal | Event rates were determined based on Chi-square goodness-of-fit test. The comparative risk of an event occurring in two months was stated as odds ratio (OR).  Multiple logistic regression models were used to calculate OR adjusted by gender, age, comorbidity and social deprivation. | More hospital admissions and mobility due to heart failure occurred in winter compared to summer.  It studies seasonal variation but not higher or colder than normal temperature and health outcomes. |
| Pell et al. (1999) Seasonal variations in out of hospital cardiopulmonary arrest. *Heart*, 82(6), 680-683. | 1988-1997 (10y) | Scotland | Mortality and morbidity (cardiopulmonary arrest) | Seasonal | Prospective cohort study using the Heartstart (Scotland) database.  Univariate comparisons of  5406 arrests occurring in summer with 5484 in winter, in terms of patient characteristics, management, and survival using  Chi-squared test and Mann-Whitney U tests. Multivariate analysis of the association between season and survival following adjustment for case mix. | Only 6% of people who arrested in winter survived to discharge, compared to 8% of those who arrested in summer (odds ratio 0.77, p < 0.001).  People who arrested in winter had a poorer risk profile in that they were older, more likely  to arrest at home, less likely to have a witness, and less likely to receive defibrillation. However, after adjustment for case mix, people who arrested in winter were still 19% less likely to survive compared to those who arrested in summer.  Deaths pre-admission were significantly higher in winter (odds ratio 1.18) but in-hospital deaths were not. |
| Aubiniere-Robb (2003). Blood Pressure Response to Patterns of Weather Fluctuations and Effect on Mortality | 5y | West Scotland | Mortality and morbidity (hypertension, blood pressure) | Seasonal | Generalised estimating equations and Cox proportional hazards model were used to model the effect of monthly average weather conditions on longitudinal blood pressure (BP) and mortality respectively. | Temperature, sunshine, and rainfall have been shown to be determinants of BP changes in the population. However, only temperature response was an independent predictor of mortality |

# 2. Study cities and regions

| City/region | Local authority |
| --- | --- |
| Edinburgh | City of Edinburgh |
| Glasgow | Glasgow City |
| Aberdeen | Aberdeen City |
| Dundee | Dundee City |
| West | Argyll and Bute |
|  | East Dunbartonshire |
|  | East Renfrewshire |
|  | Inverclyde |
|  | Renfrewshire |
|  | West Dunbartonshire |
|  | North Lanarkshire |
|  | South Lanarkshire |
|  | East Ayrshire |
|  | North Ayrshire |
|  | South Ayrshire |
|  | Dumfries and Galloway |
|  | Stirling |
| East | Aberdeenshire |
|  | Angus |
|  | Perth and Kinross |
|  | Fife |
|  | West Lothian |
|  | Midlothian |
|  | East Lothian |
|  | Scottish Borders |
|  | Clackmannanshire |
|  | Falkirk |
|  | Moray |
| North | Na h-Eileanan Siar (Western Isles) [Eilean Siar*] |
|  | Orkney Islands |
|  | Shetland Islands |
|  | Highland |

# 3. Carstairs index and deprivation

## 3.1. Census variables used for the calculation of the modified Carstairs Index

| Carstairs components and corresponding census variables | | Code of variables used | | | |  |
| --- | --- | --- | --- | --- | --- | --- |
|  |  | 1981 | 1991 | 2001 | 2011 |  |
| Overcrowding: proportion of all persons living in private households with more than one person per room | Total residents in all private households | 81sas100937 | s230045 | KS0010008 | 323022 |  |
|  | Total residents in all private households with over 1 and up to 1.5 persons per room | 81sas100948 | s230047 | Provided by GROS^1^ | 323025 |  |
|  | Total residents in all private households with over 1.5 persons per room | 81sas100947 | s230048 | Provided by GROS^1^ | 323018 |  |
| Unemployment: proportion of economically active persons seeking or waiting to start work | Total economically active persons^2^ | 81sas090719 | s090013 | cs0210010 | 246 (total economically active males) +  247 (total economically active females) |  |
|  | Total unemployed economically active persons | 81sas090859 | s090019 | cs0210046 | 246 (total economically active males) +  247 (total economically active females) +  306612 (full-time work males) +  306622 (full-time work females) +  306611 (part-time work males) +  306621(part-time work females) +  306613 (self-employed males) + 306623 (self-employed females)  All excludes full-time students |  |
| Low social class: proportion of all persons in private households with an economically active head with head of household in social class IV or V | Total residents in private households with an economically active head | 81sas525409 (social class I) + 81sas525412 (social class II) + 81sas525415 (social class III-non-manual) + 81sas525418 (social class III-manual) + 81sas525421 (social class IV) + 81sas525424 (social class V) + 81sas525427 (armed forces + inadequately described) | s900002 | uv0310001 | 2084 |  |
|  | Total residents in private households with an economically active head of social class IV (semi-skilled) | 81sas525421 | s900027 | uv0310031 (Lower technical process operator) + uv0310034 (Semi-routine Service) + uv0310310036 (Semi-routine operative) + uv0310037 (Semi-routine agriculture) + uv0310039 (Semi-routine childcare) + uv0310041 (Routine sales and service) + uv0310042 (Routine production) + uv0310045 (Routine Agricultural)^3^ | 2119^4^ |  |
|  | Total residents in private households with an economically active head of social class V (unskilled) | 81sas525424 | s900032 | uv0310044 (Routine operative)^3^ | 2127^4^ |  |
| No car: proportion of all persons in all private households which do not own a car | Total residents in all private households | 81sas100937 | s210044 | KS0010008 | 314217 |  |
|  | Total residents in all private households with no car | 81sas100950 | s210045 | Provided by GROS^1^ | 314218 |  |
| Population weighting | Total residents in all private households | 81sas010044 | s010065 | KS0010008 | 136983 |  |
| Note: 1. General Register for Scotland (GROS) provided data not available online. 2. In 2001 and 2011, economic activity is applicable to those aged 16 and 74; whereas in 1981, 1991, it refers to those aged 16 and over. 3. In 2001, the data is categorised based on the National Statistics Socio-economic classification (NS-SEC). The conversion between NS-SEC and Social Class follows Brown et al. (2014). 4. In 2011 census, the NS-SEC is only given in analytic scale. Those who belongs to NS-SEC-7 and 8 are used to approximate SC-IV and SC-V. | | | | | | |

3.2. Male and total unemployment
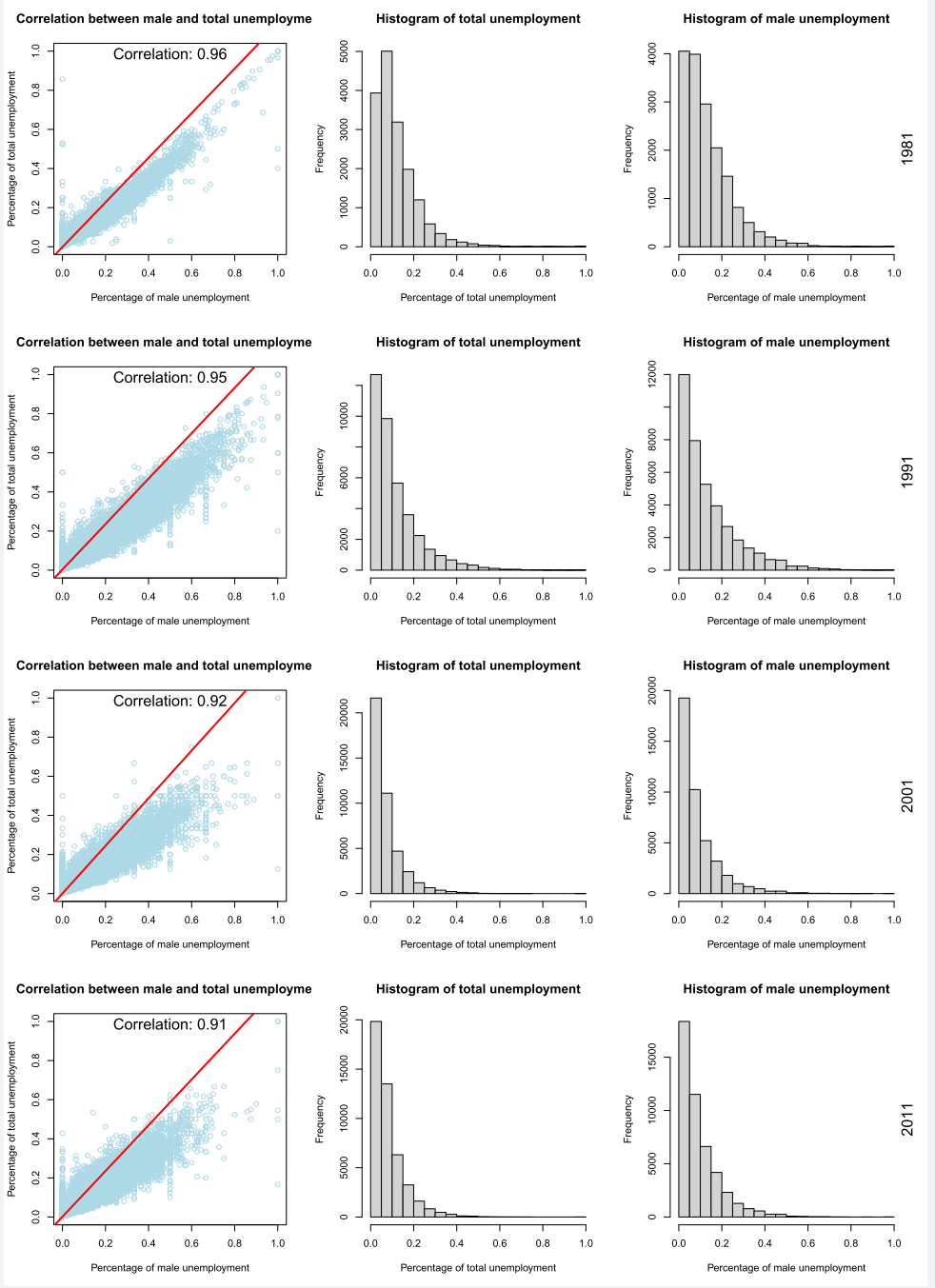


Figure A 1. Correlation and distribution of the percentage of male and total unemployment in small areas in 1981, 1991, 2001 and 2011.

## 3.3. Spatial distribution of the deprivation


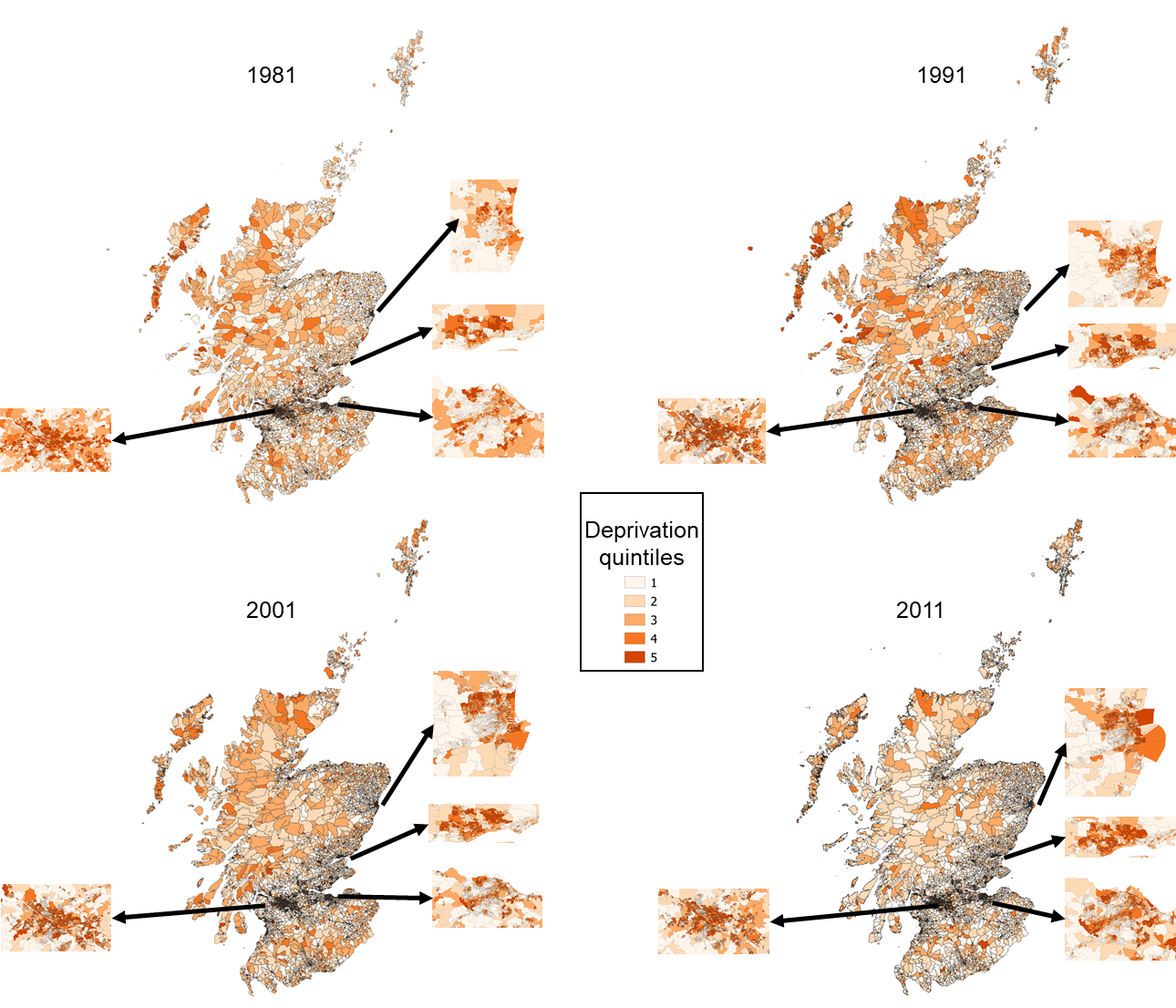


Figure A 2. Spatial distribution of deprivation quintiles in Scotland in 1981, 1991, 2001 and 2011.

# 4. International Classification of Diseases (ICD).

Table A 2. Causes of death under investigation and the associated International Classification of Diseases codes (ICD)—ICD-8, ICD-9 and ICD-10 for the 8th, 9th and10th Revision respectively.

| Time periods | 2000 onwards | 1979-1999 | 1974-1978 |
| --- | --- | --- | --- |
| Underlying cause of death | ICD-10 | ICD-9 | ICD-8 |
| All-cause | A00-Z99 | 000-799 & E800-E999 | 000-799 & E800-E999 |
| Cardiovascular diseases  (diseases of the circulatory system) | I00-I99 | 390-459 | 390-459 |
| Respiratory diseases | J00-J99 | 460-519 | 460-519 |

# 5. Sensitivity analysis

Table A 3. Model settings for sensitive analysis. Only the settings that are different from the main model are listed here.

| Description | OtA model | JJA model | Data |
| --- | --- | --- | --- |
| S1: Daily mean temperature | NS with 1 internal knot at the 50^th^ percentile, 2 internal knots at the 50^th^ and 90^th^ percentile and 3 internal knots at the 25^th^, 50^th^ and 75^th^ percentile of OtA temperature distribution. | NS with 1 internal knot at the 50^th^ percentile, 2 internal knots at the 50^th^ and 90^th^ percentile and 3 internal knots at the 25^th^, 50^th^ and 75^th^ percentile of JJA temperature distribution. | Four cities and three regions, 1974-2018 |
| S2: Maximum length of lag | 1 (dummy variables), 7 and 21 days (NS with 1 and 3 internal knots at equally-spaced log-values). | 3 days (dummy variables), 7 and 14 (NS with 1 and 2 internal knots at equally-spaced log-values). | Four cities and three regions, 1974-2018 |
| S3: Long-term trend and medium-term variation in time | 1) Quadratic and linear terms of date for long-term trend.  NS of day-of-OtA with 5df for medium-term variation.  2) NS with 5*45 df for the entire JJA dataset. | 1) Quadratic and linear terms of date for long-term trend.  NS of day-of-year with 3df for medium-term variation.  2) NS with 3*45 df for the entire JJA dataset. | Four cities and three regions, 1974-2018 |
| S4: Daily PM_10_ and ozone | NS of 2−day average value (the concentration on the same day and one day before) with 4df | | Edinburgh, 2004-2018 |
| S5: Relative humidity | NS of 2−day average value (the concentration on the same day and one day before) with 4df | | Edinburgh, 2004-2018 |

Results of sensitivity analysis:


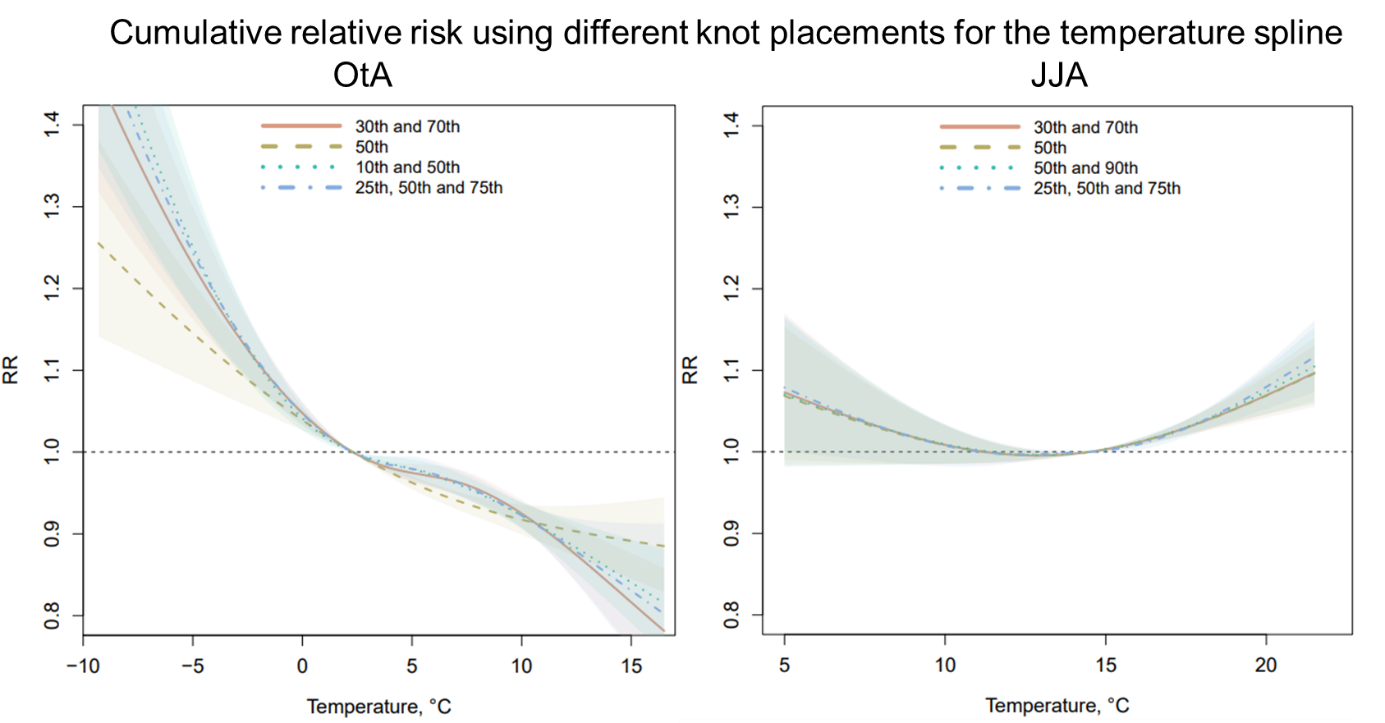


Figure A 3. Sensitivity analysis 1: natural cubic spline of daily mean temperature.


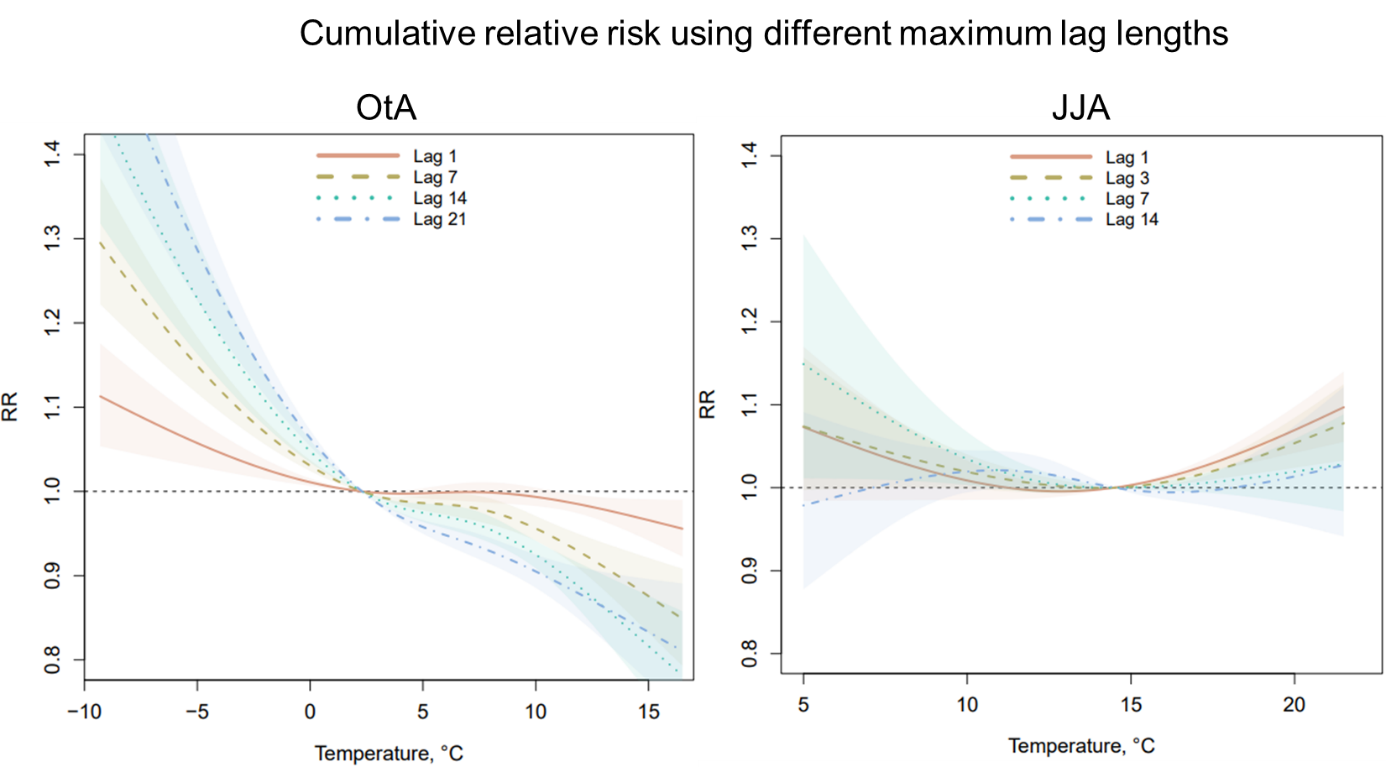


Figure A 4. Sensitivity analysis 2: maximum length of lag.


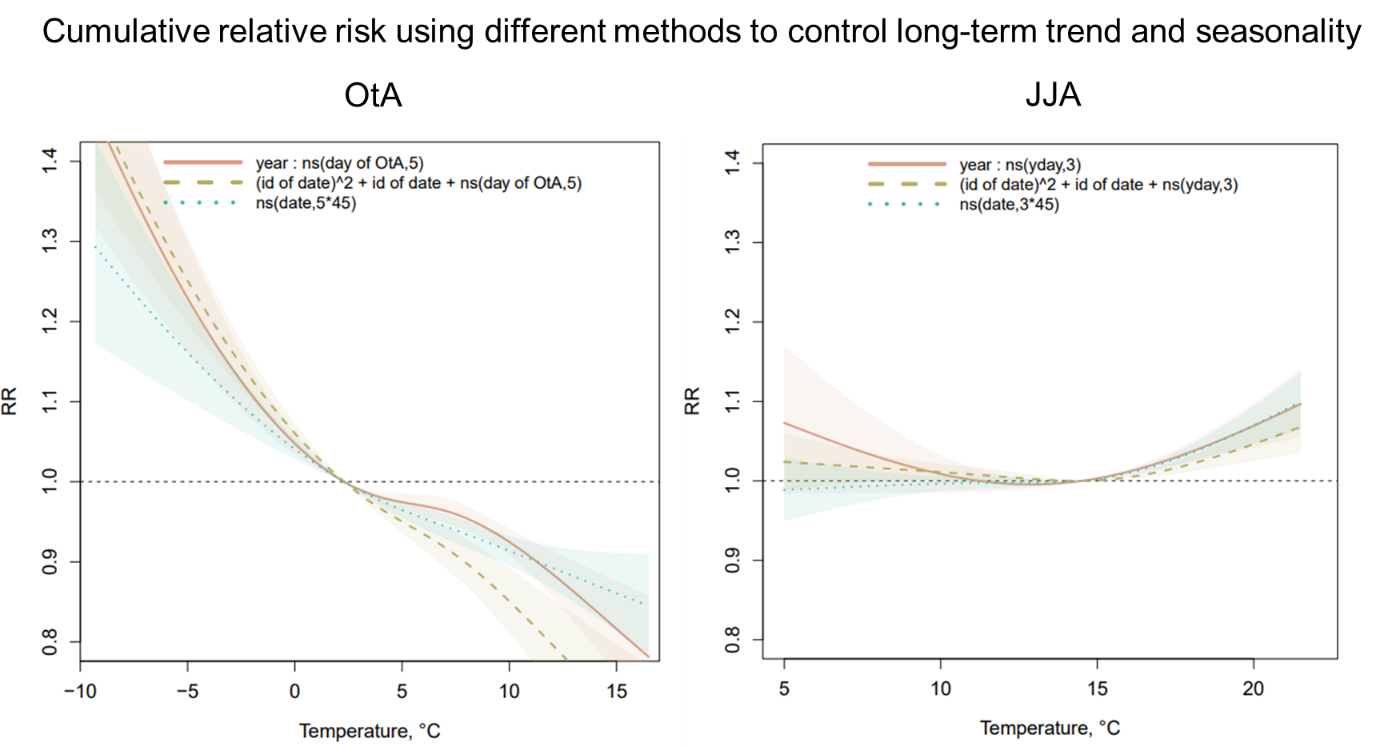


Figure A 5. Sensitivity analysis 4: control of long-term trend and medium-term variation in time.


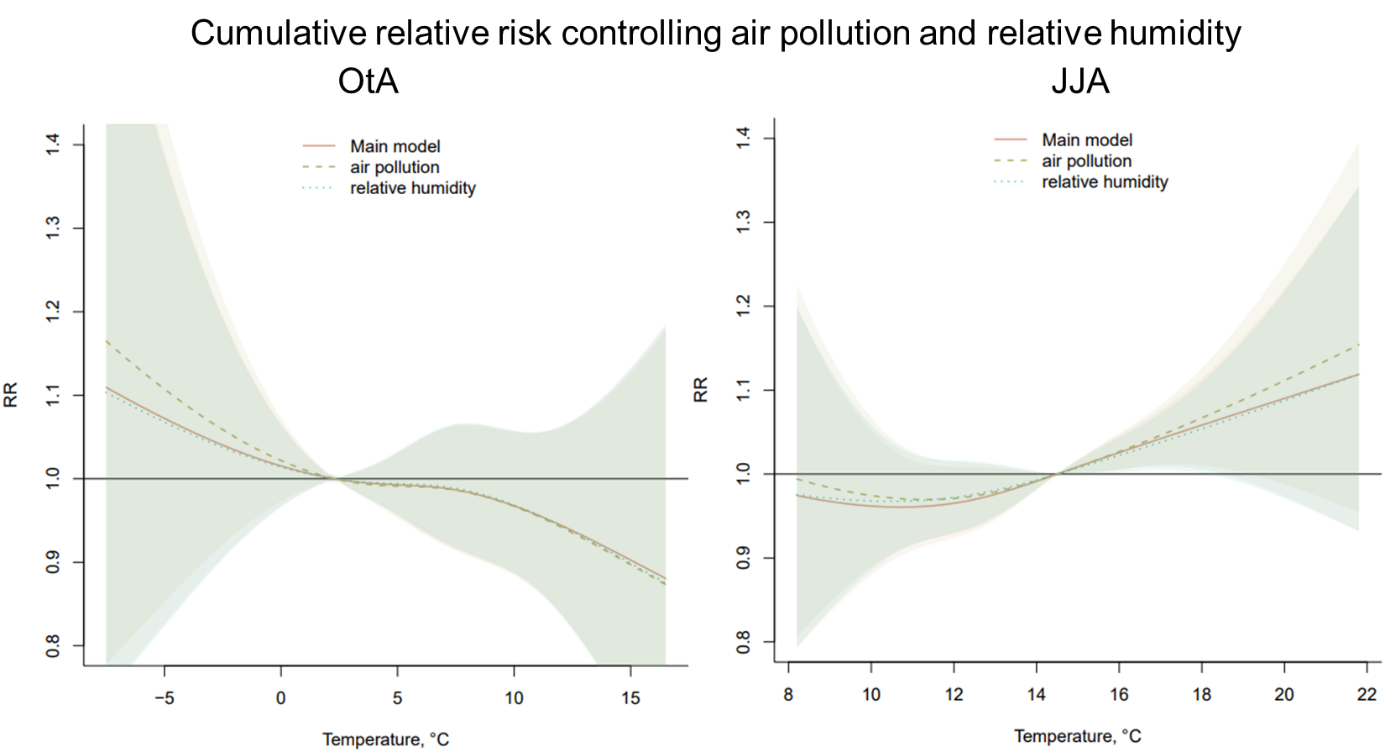


Figure A 6. Sensitivity analysis 4 and 5: control of air pollution and relative humidity.

# 6. Descriptive statistics

Table A 4. Daily mean temperature and median mortality in each month in each city and region.

|  | Daily median mortality count and 10^th^ and 90^th^ percentile in brackets | | | | | | | | | | | |
| --- | --- | --- | --- | --- | --- | --- | --- | --- | --- | --- | --- | --- |
|  | Month | | | | | | | | | | | |
| Region | 1 | 2 | 3 | 4 | 5 | 6 | 7 | 8 | 9 | 10 | 11 | 12 |
| Aberdeen | 7 (4,11) | 7 (4,11) | 7 (4,10) | 6 (3,10) | 6 (3,9) | 6 (3,9) | 6 (3,9) | 5 (3,9) | 6 (3,9) | 6 (3,9) | 6 (3,10) | 7 (4,11) |
| Dundee | 6 (3,10) | 6 (3,9) | 5 (2,9) | 5 (2,8) | 5 (2,8) | 5 (2,8) | 5 (2,8) | 4 (2,8) | 5 (2,8) | 5 (2,8) | 5 (2,8) | 6 (3,9) |
| Edinburgh | 16 (10,23) | 15 (10,22) | 15 (9,21) | 14 (9,20) | 13 (8,18) | 12 (8,17) | 12 (8,17) | 12 (8,17) | 12 (8,18) | 13 (8,18) | 14 (9,20) | 15 (10,22) |
| Glasgow | 27 (18,39) | 25 (17,38) | 24 (16,36) | 23 (15,33) | 23 (15,32) | 21 (14,29) | 21 (14,29) | 20 (13,29) | 21 (14,30) | 22 (14,31) | 23 (15,33) | 26 (16,38) |
| North | 10 (6,15) | 9 (6,14) | 9 (6,13) | 9 (5,13) | 8 (5,12) | 8 (5,12) | 8 (5,12) | 8 (5,12) | 8 (5,12) | 8 (5,12) | 9 (5,13) | 9 (6,14) |
| West | 68 (56,84) | 64 (53,77) | 63 (51,75) | 59 (49,70) | 57 (47,67) | 55 (46,65) | 53 (43,63) | 53 (43,63) | 54 (45,64) | 56 (47,67) | 59 (49,71) | 65 (53,80) |
| East | 54 (43,66) | 50 (41,61) | 49 (39,58) | 47 (38,56) | 44 (35,52) | 42 (34,51) | 41 (32,50) | 40 (32,50) | 42 (34,51) | 43 (35,54) | 45 (37,56) | 50 (40,63) |
|  | Daily mean temperature and 10^th^ and 90^th^ percentile in brackets | | | | | | | | | | | |
| Aberdeen | 3.3 (0.2,6.7) | 3.6 (0.3,7) | 5 (1.7,8.5) | 6.8 (3.4,10.1) | 9.3 (6.2,12.6) | 12 (8.9,15.1) | 14.2 (11.5,16.9) | 14 (11.4,16.9) | 11.9 (9,14.9) | 9.1 (5.5,12.2) | 5.8 (2.4,9.3) | 3.8 (0,7.5) |
| Dundee | 3.4 (0.1,6.8) | 3.9 (0.5,7.4) | 5.5 (2.4,8.9) | 7.6 (4.4,10.9) | 10.4 (7.3,13.6) | 13.2 (10.4,16.1) | 15.1 (12.6,17.8) | 14.9 (12.3,17.6) | 12.6 (9.7,15.4) | 9.5 (6,12.6) | 6 (2.3,9.5) | 3.9 (-0.1,7.8) |
| Edinburgh | 3.5 (-0.3,7.3) | 3.7 (0,7.4) | 5.4 (2,8.8) | 7.4 (3.8,10.8) | 10.2 (6.9,13.5) | 13 (10.1,16) | 14.8 (12.3,17.5) | 14.6 (12.1,17.3) | 12.4 (9.4,15.2) | 9.4 (5.7,12.8) | 6 (2.1,9.7) | 3.9 (-0.4,8.1) |
| Glasgow | 3.9 (0.1,7.7) | 4.2 (0.5,7.8) | 5.8 (2.6,9.1) | 8.1 (4.7,11.2) | 11 (7.9,14.5) | 13.7 (10.9,16.8) | 15.4 (12.8,18.5) | 15 (12.6,17.8) | 12.7 (9.8,15.4) | 9.7 (6,13) | 6.3 (2.3,10) | 4.3 (-0.1,8.5) |
| North | 3.1 (-0.5,6.8) | 3.3 (-0.3,6.7) | 4.7 (1.6,7.9) | 6.9 (3.5,9.9) | 9.8 (6.7,13.3) | 12.4 (9.6,15.4) | 14.1 (11.6,17.1) | 13.8 (11.4,16.5) | 11.6 (8.7,14.2) | 8.7 (5.2,11.9) | 5.6 (1.8,9) | 3.6 (-0.4,7.7) |
| West | 2.5 (-1,6.2) | 2.8 (-0.8,6.3) | 4.3 (1,7.8) | 6.5 (3,9.7) | 9.3 (6,12.7) | 12 (9.1,15.2) | 13.9 (11.3,16.8) | 13.6 (11.1,16.4) | 11.4 (8.3,14.2) | 8.4 (4.9,11.6) | 5.1 (1.4,8.7) | 3 (-1.1,7) |
| East | 3.7 (0.7,6.5) | 3.6 (0.5,6.5) | 4.6 (1.7,7.4) | 6.2 (3.2,8.9) | 8.6 (5.8,11.5) | 10.9 (8.5,13.3) | 12.7 (10.6,15.1) | 12.8 (10.8,14.9) | 11 (8.5,13.3) | 8.6 (5.5,11.2) | 5.9 (2.8,8.7) | 4.2 (0.9,7.4) |

Table A 5. The total number of daily mortality count of subgroup in all cities and regions during 1974-2018.

| Attribute | Subgroup | Total mortality in 1974-2018 | | | | | | |
| --- | --- | --- | --- | --- | --- | --- | --- | --- |
|  |  | Abe | Dun | Edi | Gla | North | West | East |
| age | 0 to 64 | 21963 | 19324 | 46513 | 101575 | 30926 | 223619 | 153231 |
|  | 65 to 74 | 23839 | 20575 | 49908 | 98921 | 31981 | 234057 | 172437 |
|  | 75 to 84 | 33762 | 27515 | 72819 | 116339 | 46137 | 305807 | 243349 |
|  | 85 and above | 25419 | 19186 | 59232 | 71859 | 37800 | 210954 | 184838 |
| sex | Female | 55008 | 45228 | 122097 | 198615 | 72804 | 501300 | 387182 |
|  | Male | 49975 | 41372 | 106375 | 190079 | 74040 | 473137 | 366673 |
| marstat^1^ | Married | 40719 | 33045 | 83710 | 137574 | 56727 | 394521 | 309285 |
|  | Unmaried | 64264 | 53555 | 144762 | 251120 | 90117 | 579916 | 444570 |
| CoD^2^ | CVD^3^ | 43885 | 35241 | 96482 | 157371 | 63764 | 418268 | 322460 |
|  | RESP^4^ | 12529 | 11119 | 26467 | 53486 | 14936 | 116676 | 87386 |
|  | Other | 48569 | 40240 | 105523 | 177837 | 68144 | 439493 | 344009 |
| deprivation | Quintile 1^5^ | 24192 | 10407 | 53760 | 17636 | 22035 | 154573 | 137109 |
|  | Quintile 2 | 17791 | 11624 | 45303 | 29650 | 44867 | 172541 | 185136 |
|  | Quintile 3 | 26837 | 16266 | 45721 | 60381 | 42360 | 208862 | 191123 |
|  | Quintile 4 | 22420 | 22805 | 45200 | 82603 | 27017 | 232099 | 157814 |
|  | Quintile 5^6^ | 12871 | 25388 | 38184 | 198002 | 9854 | 205044 | 79751 |
|  | Missing | 872 | 110 | 304 | 422 | 711 | 1318 | 2922 |
| Note: 1. marstat: marital status. 2. CoD: Cause of Death. 3. CVD: Cardiovascular diseases. 4. RESP: Respiratory diseases. 5. Quintiles 1: the least deprived neighbourhood. 5. Quintiles 5: the most deprived neighbourhood. | | | | | | | | |

Table A 6. The total number of daily mortality count of subgroup interactions in all cities and regions during 1974-2018.

|  |  | Attribute | | | | | | | | | | | | | | | |
| --- | --- | --- | --- | --- | --- | --- | --- | --- | --- | --- | --- | --- | --- | --- | --- | --- | --- |
|  |  | age | | | | sex | | marstat | | CoD | | | deprivation | | | | |
|  | Sub-group | 0-64 | 65-74 | 75-84 | 85+ | F | M | MA | UM | CVD | RESP | Other | Q1 | Q2 | Q3 | Q4 | Q5 |
| age | 0-64 | 601980 | NA | NA | NA | 231592 | 370388 | 330534 | 271446 | 188489 | 375135 | 38356 | 73330 | 93202 | 117893 | 137575 | 173276 |
|  | 65-74 | NA | 634338 | NA | NA | 274656 | 359682 | 344086 | 290252 | 278463 | 288638 | 67237 | 82418 | 111120 | 141088 | 149235 | 146354 |
|  | 75-84 | NA | NA | 847638 | NA | 456534 | 391104 | 298940 | 548698 | 396693 | 336278 | 114667 | 134387 | 166907 | 194559 | 187245 | 160709 |
|  | 85+ | NA | NA | NA | 609939 | 423354 | 186585 | 87035 | 522904 | 279180 | 227852 | 102907 | 129595 | 135710 | 138026 | 115910 | 88778 |
| sex | F | 231592 | 274656 | 456534 | 423354 | 1386136 | NA | 349249 | 1036887 | 597132 | 620150 | 168854 | 227931 | 267671 | 307600 | 298819 | 277092 |
|  | M | 370388 | 359682 | 391104 | 186585 | NA | 1307759 | 711346 | 596413 | 545693 | 607753 | 154313 | 191799 | 239268 | 283966 | 291146 | 292025 |
| marstat | MA | 330534 | 344086 | 298940 | 87035 | 349249 | 711346 | 1060595 | NA | 443962 | 512680 | 103953 | 172789 | 205593 | 234809 | 233849 | 205862 |
|  | UM | 271446 | 290252 | 548698 | 522904 | 1036887 | 596413 | NA | 1633300 | 698863 | 715223 | 219214 | 246941 | 301346 | 356757 | 356116 | 363255 |
| CoD | CVD | 188489 | 278463 | 396693 | 279180 | 597132 | 545693 | 443962 | 698863 | 1142825 | NA | NA | 182619 | 223279 | 257376 | 247959 | 223073 |
|  | RESP | 375135 | 288638 | 336278 | 227852 | 620150 | 607753 | 512680 | 715223 | NA | 1227903 | NA | 189798 | 226023 | 263397 | 269743 | 272092 |
|  | Other | 38356 | 67237 | 114667 | 102907 | 168854 | 154313 | 103953 | 219214 | NA | NA | 323167 | 47313 | 57637 | 70793 | 72263 | 73952 |
| dep. | Q1 | 73330 | 82418 | 134387 | 129595 | 227931 | 191799 | 172789 | 246941 | 182619 | 189798 | 47313 | 419730 | NA | NA | NA | NA |
|  | Q2 | 93202 | 111120 | 166907 | 135710 | 267671 | 239268 | 205593 | 301346 | 223279 | 226023 | 57637 | NA | 506939 | NA | NA | NA |
|  | Q3 | 117893 | 141088 | 194559 | 138026 | 307600 | 283966 | 234809 | 356757 | 257376 | 263397 | 70793 | NA | NA | 591566 | NA | NA |
|  | Q4 | 137575 | 149235 | 187245 | 115910 | 298819 | 291146 | 233849 | 356116 | 247959 | 269743 | 72263 | NA | NA | NA | 589965 | NA |
|  | Q5 | 173276 | 146354 | 160709 | 88778 | 277092 | 292025 | 205862 | 363255 | 223073 | 272092 | 73952 | NA | NA | NA | NA | 569117 |

# 7. Relative risk at the 1^st^ and 99^th^ percentile of daily temperature distribution.

Table A 7. Relative risk at the 1st and 99th percentile of daily temperature distribution compared to the 10^th^ and 90^th^ percentile for the cold and heat effect respectively (95% confidence interval in brackets).

| Region | Relative risk | |
| --- | --- | --- |
|  | Cold | Heat |
| Aberdeen | 1.12 (1.04,1.20) | 1.04 (0.99,1.11) |
| Dundee | 1.05 (0.96,1.14) | 1.04 (0.99,1.09) |
| Edinburgh | 1.13 (1.08,1.18) | 1.06 (1.03,1.09) |
| Glasgow | 1.11 (1.07,1.15) | 1.04 (1.03,1.06) |
| North | 1.05 (0.99,1.11) | 0.99 (0.95,1.03) |
| West | 1.08 (1.06,1.10) | 1.04 (1.02,1.06) |
| East | 1.15 (1.10,1.20) | 1.02 (0.97,1.07) |
| Meta-estimation in cities | 1.11 (1.06,1.16) | 1.05 (1.00,1.09) |
| Meta-estimation in regions | 1.09 (1.05,1.13) | 1.03 (0.98,1.07) |
| Meta-estimation in all cities and regions | 1.10 (1.07,1.13) | 1.04 (1.02,1.05) |

Table A 8. Meta-estimation of RR among subgroups at the 1^st^ and 99^th^ percentile of daily temperature distribution for cold effects in October to April (next year, OtA) and heat effects in June, July and August (JJA) (95% confidence interval in brackets).

| Attribute | Subgroup | Meta-estimation of relative risk | |
| --- | --- | --- | --- |
|  |  | OtA | JJA |
| age | 0 to 64 | 1.06 (1.03,1.09) | 1.03 (1.01,1.05) |
|  | 65 to 74 | 1.09 (1.06,1.12) | 1.03 (1.00,1.05) |
|  | 75 to 84 | 1.10 (1.07,1.12) | 1.04 (1.02,1.05) |
|  | 85 + | 1.14 (1.10,1.17) | 1.06 (1.03,1.08) |
| sex | Female | 1.09 (1.07,1.11) | 1.04 (1.03,1.06) |
|  | Male | 1.10 (1.08,1.13) | 1.03 (1.02,1.05) |
| marstat | Married | 1.10 (1.07,1.12) | 1.03 (1.01,1.04) |
|  | Unmaried | 1.09 (1.07,1.11) | 1.04 (1.03,1.06) |
| CoD | CVD | 1.12 (1.09,1.14) | 1.03 (1.01,1.05) |
|  | RESP | 1.17 (1.13,1.22) | 1.09 (1.06,1.12) |
|  | Other | 1.04 (1.02,1.06) | 1.03 (1.01,1.04) |
| deprivation | Quintile 1 | 1.11 (1.07,1.15) | 1.05 (1.02,1.08) |
|  | Quintile 2 | 1.09 (1.05,1.12) | 1.03 (1.00,1.05) |
|  | Quintile 3 | 1.06 (1.03,1.09) | 1.03 (1.01,1.05) |
|  | Quintile 4 | 1.09 (1.06,1.12) | 1.03 (1.01,1.05) |
|  | Quintile 5 | 1.11 (1.08,1.14) | 1.04 (1.02,1.06) |
| Age*sex | age0-74_F | 1.08 (1.04,1.12) | 1.03 (1.00,1.05) |
|  | age0-74_M | 1.09 (1.06,1.12) | 1.03 (1.01,1.05) |
|  | age75+_F | 1.10 (1.07,1.13) | 1.05 (1.03,1.07) |
|  | age75+_M | 1.15 (1.11,1.19) | 1.03 (1.01,1.06) |
| Age*marstat | age0-74_MA | 1.09 (1.06,1.12) | 1.02 (1.00,1.04) |
|  | age0-74_UM | 1.08 (1.05,1.12) | 1.03 (1.01,1.06) |
|  | age75+_MA | 1.14 (1.10,1.19) | 1.03 (1.00,1.06) |
|  | age75+_UM | 1.11 (1.09,1.14) | 1.05 (1.03,1.07) |
| Age*deprivation | age0-74_Q1 | 1.07 (1.01,1.14) | 1.01 (0.97,1.06) |
|  | age0-74_Q2-4 | 1.07 (1.03,1.12) | 1.02 (0.99,1.06) |
|  | age0-74_Q5 | 1.11 (1.07,1.15) | 1.04 (1.02,1.07) |
|  | age75+_Q1 | 1.13 (1.08,1.18) | 1.06 (1.02,1.00) |
|  | age75+_Q2-4 | 1.13 (1.09,1.17) | 1.04 (1.01,1.08) |
|  | age75+_Q5 | 1.11 (1.06,1.16) | 1.05 (1.02,1.08) |
| Age* CoD | age0-74_CIRC | 1.11 (1.08,1.15) | 1.01 (0.98,1.04) |
|  | age0-74_RESP | 1.24 (1.16,1.32) | 1.11 (1.05,1.16) |
|  | age0-74_OTHR | 1.03 (1.00,1.06) | 1.03 (1.01,1.05) |
|  | age75+_CIRC | 1.14 (1.11,1.18) | 1.05 (1.02,1.07) |
|  | age75+_RESP | 1.16 (1.1,1.22) | 1.08 (1.04,1.12) |
|  | age75+_OTHR | 1.06 (1.03,1.1) | 1.03 (1.01,1.05) |
| *Note: see the full term of the abbreviations in Table A5 and A6.* | | | |

# 8. Energy performance and deprivation

The energy efficiency of domestic houses is indicated by the Energy Performance Certificate (EPC) score, which is downloaded from the Scottish Government from <https://statistics.gov.scot/data/domestic-energy-performance-certificates>. An EPC is produced when a new building has been constructed and when a new building is to be sold or rented to a new tenant. The EPC data provided by the Scottish Government is quarterly data from 2012 to 2021, and EPC scores are provided on postcode level. Therefore, there can be multiple EPC scores for each postcode. In the analysis, an average of the EPC scores between 2012 and 2021 was taken for each postcode. It was further averaged on census output levels for the comparison of EPC scores and Carstairs scores (in 2011), as shown in Figure A7. A higher EPC score indicates better energy efficiency, and a higher Carstairs score indicates higher deprivation (Pearson correlation coefficient: 0.29). Therefore, Figure A7 shows that people living in more deprived areas have tend to a higher home energy efficiency.


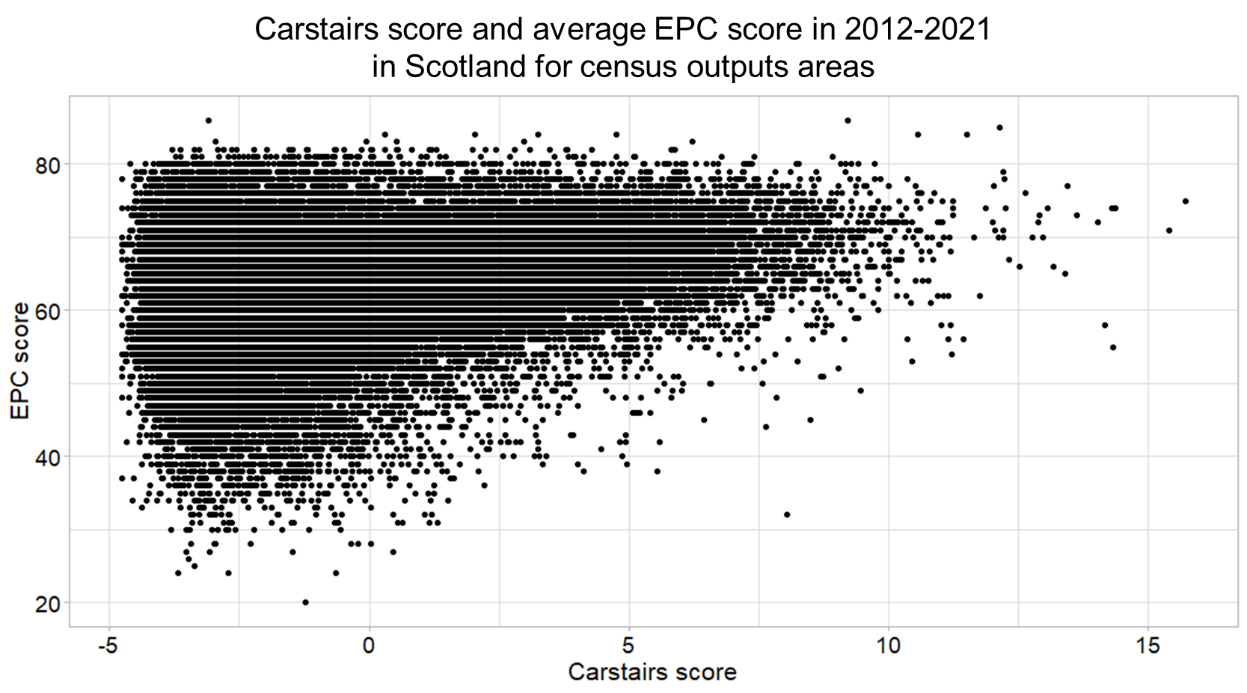


Figure A 7. Scatter plot of EPC and Carstairs scores.
